# SqueezeViX-Net with SOAE: A Prevailing Deep Learning Framework for Accurate Pneumonia Classification using X-Ray and CT Imaging Modalities

**DOI:** 10.2174/0115734056378882250831125120

**Published:** 2025-09-11

**Authors:** N. Kavitha, B. Anand

**Affiliations:** 1 Department of Electronics and Instrumentation Engineering, Hindusthan College of Engineering and Technology, Coimbatore, Tamil Nadu, India; 2 Department of Electrical and Electronics Engineering, Hindusthan College of Engineering and Technology, Coimbatore, Tamil Nadu, India

**Keywords:** Pneumonia disease detection, Medical imaging, X-Ray image, Computed tomography (CT) images, Deep learning, Optimization, Classification

## Abstract

**Introduction:**

Pneumonia represents a dangerous respiratory illness that leads to severe health problems when proper diagnosis does not occur, followed by an increase in deaths, particularly among at-risk populations. Appropriate treatment requires the correct identification of pneumonia types in conjunction with swift and accurate diagnosis.

**Materials and Methods:**

This paper presents the deep learning framework SqueezeViX-Net, specifically designed for pneumonia classification. The model benefits from a Self-Optimized Adaptive Enhancement (SOAE) method, which makes programmed changes to the dropout rate during the training process. The adaptive dropout adjustment mechanism leads to better model suitability and stability. The evaluation of SqueezeViX-Net is conducted through the analysis of extensive X-ray and CT image collections derived from publicly accessible Kaggle repositories.

**Results:**

SqueezeViX-Net outperformed various established deep learning architectures, including DenseNet-121, ResNet-152V2, and EfficientNet-B7, when evaluated in terms of performance. The model demonstrated better results, as it performed with higher accuracy levels, surpassing both precision and recall metrics, as well as the F1-score metric.

**Discussion:**

The validation process of this model was conducted using a range of pneumonia data sets, comprising both CT images and X-ray images, which demonstrated its ability to handle modality variations.

**Conclusion:**

SqueezeViX-Net integrates SOAE technology to develop an advanced framework that enables the specific identification of pneumonia for clinical use. The model demonstrates excellent diagnostic potential for medical staff through its dynamic learning capabilities and high precision, contributing to improved patient treatment outcomes.

## 1 INTRODUCTION

Pneumonia is a major respiratory tract infection that often affects large populations, particularly in developing and underdeveloped countries, where socio-economic problems increase the prevalence and severity of this infection [1]. It is essentially an infection caused by the invasion of certain bacteria or viruses into the lungs, which then diffusely causes inflammation and edema of the alveoli—small, air-sac-like structures used for the exchange of oxygen. These are the organisms responsible for infections that lead to a subsequent immune response, characterized by fluid leakage, or pleural effusion, within the lung tissues. It is an acute pulmonary infection that affects the overall functioning of the lungs. If not diagnosed and treated on time, it may lead to the potential development of serious health problems [2, 3]. It primarily develops through an attack by various pathogens, such as bacteria, viruses, or fungi, which are capable of causing inflammation or infection in the lungs. Within the lungs, they fill the small air sacs, or alveoli, with pus and fluid, resulting in a painful condition known as pleural effusion. Normal aeration of the fluid is limited in nature, leading to labored breathing, chest pain, and other respiratory complications that may differ in individuals based on age and patient immunity [4]. It is, in particular, among the leading causes of death in young, especially under-five-year-old children, and is considered to be responsible for more than 15% deaths in this tender age group [5, 6]. Thus, the high mortality among children urgently requires identification and intervention to prevent rapid complications of this infection into severe respiratory distress and other life-threatening situations. Pneumonia is always an uphill task in underdeveloped and developing nations due to the fact that the prevalence of the disease is heightened by environmental and socio-economic aspects [7]. Additionally, besides good healthcare access, poor medical facilities and a lack of infrastructure make timely diagnosis and treatment difficult. All these reasons make a larger chunk of the population susceptible to this disease. Under such conditions, the chances of developing pneumonia increase; hence, the children among these are more prone to take a toll due to poor immunity. These are just some of the challenges; thus, the a need for diagnostic techniques that are cheaper and more easily accessible in poor-resource areas. Radiological imaging is one of the primary bases for the early detection and management of pneumonia, thereby reducing its burden and preventing fatalities. It is among the most effective techniques for diagnosing pneumonia and maintaining lung health, alongside other imaging techniques such as CT, MRI, and radiography. Of all these, a chest X-ray is preferred as the diagnostic tool due to its nature of being non-invasive and reasonable in cost compared to the rest. X-rays detect fluid and inflammation of lungs that are typical for pneumonia [8, 9]. This, therefore, means that the early diagnosis due to the X-rays can mean the doctor starts the treatment immediately. As said above, X-rays are very important, particularly in resource constraint regions in view of their quick and supportive diagnosis that leads to early treatment. Due to the fact that pneumonia is a progressive infection with serious health implications, early radiological detection through the use of an X-ray examination will reduce complications and aid life-care. This is important because early diagnosis of pneumonia can be treated making a huge difference to a patient’s results reducing the likelihood of further adversities or even death. Since early pneumonia may be barely distinguishable and left untreated or treated later may reach its advanced stages quickly, much stress is put on the respiratory system and the patient’s body as a whole.

Early identification of pneumonia can determine the difference between a controllable infection and a deadly disease, which plays a role in cost-effective diagnostics [10]. Chest X-ray is the most common diagnostic technique for diagnosing pneumonia because it is accurate and reasonably inexpensive to produce an image that shows signs of infection in the lungs. A chest x-ray enables the identification of typical patterns of fluid accumulation and inflammation, as well as specific structural changes that accompany pneumonia, facilitating a correct diagnosis. X-ray imaging is particularly beneficial in situations where resources are limited, as it is cheaper and more widely available than imaging techniques such as computed tomography or magnetic resonance imaging scans, which may not be accessible in rural or low-resource hospitals [11, 12]. Chest X-rays, therefore, have emerged as a crucial way of diagnosing pneumonia worldwide and persuasively treating the infection, as early detection is highly key and life-determining for patients in vulnerable groups or countries. The development of an automated system for pneumonia detection is crucial, as the desired outcome of faster and better pneumonia diagnosis can only be achieved if the utilization of resources and trained radiologists is a problem in the region most in need. In this current work, we developed a CAD system for characterizing CXR images using pre-trained deep transfer learning models to classify the images accurately. CAD systems [13] have been used to assist healthcare professionals by analyzing medical images and providing accurate diagnostic information. Typically, the CAD system will be enhanced with deep learning, a higher-order subset of AI, which effectively emulates the human cognitive process. It will enable further improvements in detection capability by learning from the complex patterns of chest X-rays indicative of infection [14].

The pneumonia epidemiological data and trends indicate that they vary from one region and population group to another. Regarding the differences in healthcare settings, socio-economic status, environmental conditions, and vaccination coverage, it is known that there is enormous variation. Pneumonia ranks among the leading causes of morbidity and mortality worldwide, particularly among children aged under five years and the elderly. For instance, a WHO (World Health Organization) estimate suggests that almost 15% of the deaths of children under five years are due to pneumonia. In LMICs such as sub-Saharan Africa and South Asia, incidence and death rates of pneumonia are relatively higher because access to health facilities, delays in diagnosis, and low use in terms of preventive measures, such as immunization and improved nutrition, contribute to the high rates. Contrastingly, the rich regions have managed to lower pneumonia-related deaths due to better healthcare systems, advanced access to imaging technologies, and comprehensive vaccination programs. Nevertheless, pneumonia poses health threats to immuno-compromised individuals, chronic disease patients, and the elderly in these high-income countries. Seasons, as well as environmental factors such as air pollution and smoking, account for the variations in pneumonia trends across different geographic zones.

Thus, automation provides a means for effectively diagnosing pneumonia, saving time and money. These, therefore, will be useful in resource-constrained settings where early intervention can be made to prevent an infection from worsening, hence reducing mortality rates. This work also leverages the power of system design by developing an ensemble of transfer learning models to further enhance robustness and accuracy through multiple model perspectives, thereby increasing the reliability of classification results. Deep learning is one of the premier foundational technologies of AI and is notable for its performance in solving complex tasks in computer vision [15, 16]. Large deep learning models have been extensively used in various industries for image classification tasks in recent years, as they can handle the automatic learning and recognition of very small patterns in data, often more effectively than traditional machine learning methods. In specific applications, such as biomedical image classification, obtaining this huge amount of labeled data is challenging, as expert physicians must label each image. That process is expensive, time-consuming, and labor-intensive, as the task demands expertise from well-trained radiologists or experts to guarantee the correctness of the data annotations and their clinical validity. Therefore, in diseases for which rapid and accurate diagnosis is very crucial, such as pneumonia, one of the biggest deterrents to the large-scale application of deep learning has been the limited availability of large labeled datasets.

In practice, this limit can be circumvented by transfer learning: effective deep model development is possible even when limited medical data is available. Transfer learning reuses a model that was pre-trained on a large dataset for some general image classification problem, such as another problem with a smaller dataset—for instance, classifying chest X-rays for pneumonia diagnosis. The weights of a pre-trained model provide a useful starting point that enables the model to adapt to the task at hand, thereby requiring significantly less new data. Knowledge transfer, in other words, involves moving from the original large dataset's starting points toward solving the latest problem, updating itself to fit the peculiarities of the smaller dataset. Indeed, this has proven to be a highly effective strategy, finding widespread adoption in biomedical image analysis, given the general scarcity of high-quality labeled data [16, 17]. Transfer learning provides a mechanism for developing our CAD system to leverage strengths from well-trained models and tune them to perform reliably on chest X-ray data for pneumonia classification, thereby bypassing the typical data bottleneck that restrains deep learning applications in medical practice.

In ensemble learning, the predictions from several models or classifiers are combined to yield a single, more accurate prediction for a given test sample. The method of ensemble learning integrates the decisions of several models to capture a wider range of discriminative information than any individual model can achieve. This helps improve the general predictive performance, as it leverages the unique strengths and perspectives of each classifier, thereby reducing the risk of error due to reliance on a single model [18]. In practice, ensemble learning, when applied to data classification, typically combines all the predictions generated by each base classifier and synthesizes them into one cohesive prediction, yielding a result that is normally more reliable and closer to accuracy. This technique has a wide range of applications across various cases where high precision is required, as it maximizes the system's ability to make accurate classifications across diverse samples. A variety of ensemble methods have been developed, differing in how the base classifiers predict their combination. The most commonly used techniques are average probability, weighted average probability, and majority voting [19, 20]. The average probability approach is simpler in that all models in the ensemble hold equal priorities; the probabilities that each classifier provides for a class are averaged, and the highest averaged probability becomes the final prediction. While the approach works very well in applications, it assumes that all the classifiers are equally good at capturing the relevant features of the data. The reality is that for the detection of certain patterns or features, there might be better models and thus ones that could serve more reliably for certain predictions.

The sophisticated methods include, for example, the weighted average probability of every classifier in the ensemble. In this, every output of the prediction of a classifier is multiplied by a previously assigned a priori weight reflecting the classifier's relative importance or expertise on the problem at hand. Weighted methods generally enjoy far more flexibility and adaptiveness compared to the equal-weight strategy, as they take into consideration the issue of competency difference among the various classifiers [21]. However, the optimum performance of the ensemble would depend significantly on the choice of an appropriate weight for each classifier. In fact, this determination is rather delicate, balancing the strengths which each classifier offers against the overall consistency and reliability of the ensemble. If successful, weighted ensembles can yield a significant boost in predictive accuracy by leveraging the strengths of the base classifiers collectively, thereby providing a robust and versatile solution to the problem at hand. It is against this urgent need for improvement in diagnostics of pneumonia, coming with a substantially higher level of accuracy and speed while ensuring access even to resource-poor settings, that the motivation for developing a hybridized deep optimized learning system for the classification of pneumonia by using both CT and X-ray images is established [22, 23]. However, classic diagnostic techniques are effective yet somewhat limited in some respects. X-ray imaging is widely available and relatively inexpensive, but it does not always provide the detailed information necessary for highly accurate diagnosis, particularly in complex cases. CT imaging provides a more precise view of lung structures, where changes due to pneumonia can be more clearly visible; however, it is often more expensive, time-consuming, and less accessible in many healthcare settings. Such a hybridized framework, which exploits both types of imaging, can provide a much more comprehensive and robust analysis, thereby enabling detection with higher precision in a wide variety of cases.

While deep learning models have demonstrated phenomenal performance in medical imaging, most of these models are sensitive to the quality and quantity of the training dataset and struggle to generalize well to diverse or complex datasets. On the other hand, pneumonia classification is a diverse disease presentation, requiring a model architecture that is highly adaptable and can handle variations in image type, quality, and resolution. The challenge faced in such instances motivates the incorporation of optimized learning techniques that augment the robustness and efficiency of a model [24]. Hybridizing deep learning with optimization techniques enables the system to dynamically adjust its parameters, resulting in high-performance models that are applicable to heterogeneous datasets. Incorporating optimization will hence allow the model to learn more effectively from the CT and X-ray images, ultimately leading to a system that can handle variations in image data. This is further inherent in the limitation of individual imaging modalities and models. Both CT and X-ray images provide different information, and their combined model will have more diverse diagnostic features in processing. The use of deep learning in the proposed methodology enables the automatic learning of complex features from images. At the same time, optimization ensures that such learning is effective, fast, and responsive to real-world constraints [25, 26]. Such hybridized, optimized learning may be able to achieve more than just increased accuracy in pneumonia classification; it may also enable its wide applicability, especially where rapid yet reliable diagnosis can make the difference between life and death. In all, this work aims to devise a model that addresses the current gaps in pneumonia diagnosis and develops a robust, versatile, high-performance solution suitable for deployment in well-equipped hospitals as well as under-resourced clinics.

To make our model robust and generalizable across different epidemiological profiles, it is essential to apply this model to a dataset as diverse as the pneumonia images collected from multiple sources and populations. The inclusion of both X-rays and CT scans across different age groups, genders, and clinical profiles enables SqueezeViX-Net to accommodate the subtle yet critical differences in the radiological presentation of pneumonia across demographic boundaries. This factor renders this model not only technically efficient but also clinically sound in real-life heterogeneous scenarios. By aligning the model's performance with these epidemiological trends, the proposed technique promises to help close a diagnostic gap in underserved areas of the world, as well as introduce a significantly improved clinical workflow in resource-rich settings.

This study derives its motivation from the pressing need for a highly accurate, computationally efficient model of pneumonia classification. This decision can be made across various imaging modalities, including X-rays and CT scans. Conventional models of deep learning have limitations, including static dropout mechanisms, a failure to adapt well to varied data distributions, and overfitting to training samples, which restrict their applicability in actual clinical scenarios and thus do not provide realistic outcomes for practical applications. The present work represents an inspired strategic motivation to fill this missing link: the development of a lightweight yet powerful architecture called the SqueezeViX-Net, which incorporates the element of Self-Optimized Adaptive Enhancement (SOAE). The dynamic nature of SOAE will thus allow the model to modify its dropout rate during training relative to the learning context, enhancing generalization while trading off accuracy. This new approach is reflected in the forward-looking motivation to set up a highly intelligent, even flexible, model that can handle the complexity and variability inherent in clinical diagnostics, thereby facilitating faster and more reliable decision-making in critical, life-threatening respiratory conditions such as pneumonia.

Interest in the study of automated pneumonia detection and classification is at an all-time high due to the pressing need for diagnostic tools that can be relied upon to assist in early and precise disease diagnosis. Pneumonia features among the leading causes of morbidity and mortality in the world; it especially prevails in young children and older adults [27]. Timely intervention plays a crucial role in mitigating this condition, especially since outcomes depend entirely on timing. While traditional diagnostic techniques, such as X-ray and CT, have proven efficient, the heavy reliance on radiologists' expertise results in inter-observer variability that may affect the consistency of diagnoses. In light of this, the last decade has seen the increasing use of ML and DL by researchers for fine-tuning diagnoses through automated searches for patterns of diseases in medical images, thereby reducing human errors. While many of these methods used handcrafted feature engineering, domain experts would manually identify specific image features indicative of pneumonia, such as texture or shape features, and then feed those into classifiers to derive a diagnosis.

These approaches could be quite powerful, yet their success was inherently limited by shortcomings in their handcrafted features, which may fail to model the rich variability in the visual appearance of pneumonia across diverse patient populations [28, 29]. Success has been notable with CNN-based models that capture intricate patterns and textures within chest X-rays and computed tomography images, achieving high accuracy rates for pneumonia detection, all without explicit manual feature extraction. Despite the much-anticipated progress achieved by CNNs and other DL architectures, generalization and robustness for different datasets are still in a challenging position. Several works utilize transfer learning from networks that have been pre-trained on smaller, domain-specific datasets. This is especially beneficial in medical imaging, as large, labeled datasets are often difficult to obtain due to the time-consuming and expertise-driven nature of annotation. While all these developments have improved the diagnostic accuracy of the technology, variability in imaging modalities, patient demographics, and disease presentation still poses challenges for existing models. This is, therefore, a call for hybridized, optimized approaches that can now leverage diverse image sources and promote model adaptability in clinical applications.

Despite having preventable measures, pneumonia still holds its place as one of the world's leading causes of morbidity and mortality, especially among children younger than five years, the elderly, and people with an immuno-compromised condition. However, pneumonia is preventable and treatable yet remains an aggressive killer in low- and middle-income countries due to inequities that exist between the various communities regarding access to preventive techniques, timely diagnosis, and suitable treatment. Immuni-zation plays a considerable role in reducing pneumonia incidence. Pneumococcal conjugate vaccines (PCVs), Haemophilus influenzae type b (Hib) vaccines, and seasonal influenza vaccines are the most vital pillars of any national immunization schedule; yet, coverage is uneven across the globe. In most regions, particularly sub-Saharan Africa and parts of South Asia, logistical challenges, vaccine supply issues, and socio-political barriers hinder full-scale implementation. Inadequate healthcare infrastructure, low public health spending, and poor awareness intensify and aggravate the scenario; early intervention capacity is limited, and there is an increased risk of complications or death from otherwise treatable infections.

Progress in controlling pneumonia is also under threat from several new challenges. Antimicrobial resistance (AMR) can quickly render even commonly available antibiotics ineffective, complicating and increasing the cost of treatment, especially in settings without diagnostic availability to guide therapy. Day-to-day indoor air pollution from the use of biomass fuel, smog, tobacco smoke, and poor ventilation further increases the environment-related risk of respiratory infection, especially among children from the crowded, impoverished communities. Malnutrition and concomitant diseases such as HIV, tuberculosis, and diabetes also compromise immunity. “The lockdown made things worse because immunization is disrupted, health systems are overburdened, and resourcing has been shifted away from pneumonia interventions.” Thus, a comprehensive intervention would involve universal coverage with a good vaccine, improved diagnostic technologies, antimicrobial stewardship, enhanced nutrition, clean air initiatives, and international funding, as well as harmonized health policies. This would prevent pneumonia from becoming a significant public health threat internationally.

Ibrahim * et al*. presented a deep learning-based methodology for classifying CXR images into COVID-19, non-COVID-19 viral pneumonia, bacterial pneumonia, and normal using a pre-trained model, AlexNet [30]. Their model will utilize CXR data from various public databases in binary, three-way, and four-way classifications, enabling high accuracy in distinguishing among the conditions. It was specifically trained for binary classifications between COVID-19 and normal, bacterial pneumonia and normal, non-COVID-19 viral pneumonia and normal, and COVID-19 and bacterial pneumonia, allowing the network to focus on discriminative features unique to each category. More than a binary classification task, the model was further optimized for three-way classifications where it differentiates between COVID-19, bacterial pneumonia, and normal cases, and a four-way classification distinguishing COVID-19 from bacterial pneumonia, non-COVID-19 viral pneumonia, and normal cases. The authors relied on pre-training with AlexNet to mitigate issues related to limited data availability. The model's multitasking capability can adapt to various diagnostic scenarios, which can be very useful for clinical contributions, as differentiating between COVID-19 and other forms of pneumonia is crucial for proper patient management. Overall, the method adopted by the authors is representative of the potential that pre-trained deep learning models possess for providing a multiclass, flexible diagnostic tool for CXR image analysis, contributing to rapid and accurate respiratory condition classification. Wang, *et al*. introduced a deep learning algorithm, Pneumonia-Plus, designed to classify pneumonia types into bacterial, fungal, and viral classes using computed tomography images with improved accuracy [31]. Pneumonia-plus is designed to match diagnostic capability with an experienced radiologist and minimize the chances of misdiagnosis as much as possible by reliably discriminating among types of pneumonia. These algorithms were developed to support clinicians to make an evidence-based timely decision for the enhancement of patient outcomes by quicker and precise diagnosis. This is particularly true because Pneumonia-Plus has gone a step further by making use of CT imaging, which offers the best possible detail available on lung structure, so as to outline the fine variations in the imaging characteristics according to the causative pathogens of pneumonia and thus enable diagnosis with high precision in clinical needs and patient care.

Sharma and Guleria did a systematic review of the literature on various methods of pneumonia detection using deep learning [32]. The superiority of deep learning over traditional machine learning techniques has been cited due to its high performance, which enables automatic feature extraction. Furthermore, the architecture and operation of each method are elaborated upon in the paper. Through this elaborate performance analysis, the authors have highlighted the salient features of these models in relation to the challenges posed by the medical imaging dataset for pneumonia detection. Comparisons of CNN-based, pre-trained, and ensemble model performances are presented in terms of different metrics, considering performance metrics, hyperparameter settings, and model fine-tuning. The meta-analysis, therefore, possesses the strong features of high-performance ensemble models, which can enhance diagnostic accuracy in pneumonia detection. Goyal *et al*. proposed a new framework for predicting lung diseases, including pneumonia and COVID-19, using chest X-ray images [33]. The authors utilized two publicly available chest X-ray datasets and, due to the aforementioned issues with image quality degradation of X-rays, employed a combination of median filtering and histogram equalization to enhance the image clarity. A modified region-growing technique was developed for achieving precise extraction of ROIs for chest regions by dynamically selecting the regions based on pixel intensity and then applying morphological operations to refine these regions. It also points out in the framework that disease prediction feature extraction must be robust, including the visual, shape, texture, and intensity features of each ROI, which are then normalized to maintain consistency. To this end, it adopts a holistic approach to improving the detection of lung diseases, featuring an overall efficient multi-step pipeline for dependable diagnostic support.

Sharma, *et al*. focused on how efficiently deep learning can detect pneumonia, particularly targeting the performance of the neural network model combined with the VGG16 architecture [34]. Hence, the authors proposed a deep learning-based model to support medical experts in diagnosing pneumonia using chest X-ray radiographs. The model has been designed in such a way that it can classify X-ray images into two classes: normal and pneumonia. These results are compared with the work of the NN using VGG16, as well as other machine learning models, including the VGG16-combined SVM, VGG16-combined KNN, and VGG16-combined Random Forest, on two different datasets. In concrete terms, with VGG16, deep learning significantly outperforms its classical counterpart in classification accuracy, thereby enhancing its potential for clinical reliability in the early detection of pneumonia. This research demonstrates how leveraging deep-learning models can improve accuracy in diagnosis, supporting clinicians even better. Sharma, *et al*. presented a deep learning-based model using VGG19 to classify pneumonia from normal lungs in chest X-ray images [35]. X-ray images can aid in diagnosing pneumonia and other diseases, such as COVID-19 and cancer; hence, precision in diagnosis is highly crucial, as incorrect identification may lead to severe consequences. In this work, the VGG19 model was trained on a dataset of 5,856 chest X-ray images for pneumonia classification. This paper selects VGG19 because it is one of the most established deep-learning-based CNN architectures for image recognition, which yields highly accurate results in distinguishing between different conditions of pneumonia and normal lungs. The approach will help reduce the need for a skilled radiologist in every case and provide a computationally reliable alternative for early diagnosis with accuracy, at least in locations where qualified radiologists can be in short supply.

Yi, *et al.* presented a deep convolutional neural network model that is scalable and interpretable in nature, which the researchers believe would be helpful for detecting pneumonia from chest X-ray images [36]. They attempt to create an effective tool to lighten the diagnostic load for health professionals by automating the process of detecting pneumonia, a task previously considered the domain of expert radiologists only. The model will utilize an enhanced architecture of DCNN to extract the relevant features from the X-ray images and then classify them into two distinct classes: normal and pneumonia. Therefore, it enhances effectiveness and efficiency in pneumonia diagnosis by providing an automated system for early diagnosis. These include the computation of classification accuracy, sensitivity, and specificity, each of which ensures the overall reliability of the system's trust in applications in real-world settings. It is here that the research demonstrates, through these reviews, that the proposed DCNN model can enhance both speed and accuracy in pneumonia diagnosis, resulting in improved patient outcomes, particularly in resource-constrained settings with a shortage of expert radiologists. Sanghvi, *et al*. has identified a framework that would help medical experts, specifically radiologists, in identifying COVID-19 and pneumonia by applying transfer learning methods [37]. The proposed system integrates a GUI tool in such an interactive manner that technicians can easily and with minimal effort upload CXR images. Once this is done, the software will process the image through its detection model, which is specifically designed for the diagnosis of pneumonia and COVID-19. The system, after processing, classifies the disease present in the radiographs and provides a clear diagnosis. A feedback mechanism has also been developed to assist the radiologist in decision-making, thereby enabling them to verify similar CXR images and diagnose with increased precision and speed. It would, however, be particularly useful for resource-constrained medical facilities or radiologists by providing an automated aid in clinical decisions. Adopting this framework represents a significant stride in the development of healthcare technologies, enabling faster and more accurate diagnoses for improved patient outcomes and helping clinicians manage a large volume of diagnostic work.

The discussion in this paper is therefore limited to the critical period between 2020 and 2023, during which the COVID-19 pandemic significantly impacted the world [38]. During this time, a comprehensive discussion on the shortcomings of the available methods for pneumonia detection and their relative effectiveness can be conducted. The present paper critically evaluates the applications of deep learning techniques in light of pandemic conditions, suggesting that these methods may not only serve as a supporting tool but can also potentially replace expert radiologists. This resource was stretched during the pandemic due to surging cases and a scarcity of trained professionals. The authors introduce the rationale behind the study, highlighting the need to utilize deep learning models in the medical imaging context, particularly in diseases such as pneumonia and COVID-19. They support the rationale behind the choice of certain resources and methodologies in this study and relate them to the approach used to address the challenges posed by the pandemic. This paper provides a critical analysis of existing solutions for pneumonia detection using CXR images, pinpointing the limitations inherent in these methods while offering insight into both specific case studies and broader perspectives. Among these, one of the key findings of their analysis is the transformative potential brought about by the emergence of Vision Transformers (ViT) as one of the most promising techniques in the domain of pneumonia detection. This conclusion aligns with the broader consensus within the field that the ViT opens up new and hopeful perspectives for further improvements in the accuracy and efficiency of automated pneumonia detection from CXR images. The authors hope that their contributions provide a better balance to the state of the art in the field, along with guidance for future research and development toward improved solutions.

Ibrahim *et al*. presented a new multi-classification deep learning model for the diagnosis of COVID-19, pneumonia, and lung cancer, utilizing two modalities: chest X-ray and computed tomography imaging [39]. This is, in fact, a dual-modality approach that capitalizes on the strengths of the two imaging types. Although chest X-rays are more readily available and often used in the diagnosis of lung conditions, they may fail in the early detection of diseases. This not only overcomes the shortcomings of dependence on a single type of imaging but also facilitates a wider diagnostic approach. The study has its uniqueness in the field, and the authors declare that, to date, no deep learning model has been developed with the specific purpose of distinguishing between COVID-19, pneumonia, and lung cancer using chest X-ray and CT images together. It therefore represents a novel approach that is expected to raise the bar in diagnostic performance and may offer clinicians a more effective tool for differentiating these lung diseases, each of which requires distinct therapeutic strategies. Moussaid, *et al*. introduced an advanced deep learning architecture using EfficientNetB7, a high-performance and state-of-the-art convolutional neural network, to describe a new architecture for classifying lung images from X-ray and CT scans into three categories: common pneumonia, coronavirus pneumonia, and normal cases [40]. It has been chosen for its state-of-the-art feature extraction and learning process, enabling it to address the challenging task of distinguishing subtle and overlapping features between different types of pneumonia and healthy lungs. The work is compared with various other recent techniques for pneumonia detection in terms of their accuracy in image classification, which establishes the robustness and consistency of EfficientNetB7 in medical image classification. This architecture leveraged the model's distinctive capabilities to achieve high predictive accuracy across the three categories, effectively distinguishing between common pneumonia, COVID-19 pneumonia, and normal cases in both imaging modalities. Additionally, by utilizing both X-ray and CT images, a more comprehensive dataset can be created, allowing the model to generalize better in performance across different diagnostic scenarios. It brings to the fore the necessity of implementing advanced architectures, such as EfficientNetB7, in medical imaging for enhanced early detection in support of diagnostic processes, thus reinforcing clinicians with a reliable tool for investigators who often find the identification and differentiation of respiratory diseases problematic unparalleled experience of having to face the COVID-19 pandemic.

A critical review of the literature on pneumonia detection studies using machine and deep learning approaches has identified several key gaps that require further research and innovation. First, although various studies prove the efficacy of deep learning architecture, most are bound within small datasets that suffer either from imbalanced classes or small sample sizes, which might affect the generalization capability of their results. As useful as the use of only publicly available data may be, it may fail in capturing such diversity clinically, for example, in variations within image quality, demographic factors, and disease severity. Again, this calls for larger data that will represent real-world cases across diverse patient populations and various imaging conditions. Besides, although transfer learning and ensemble models have been proposed to overcome small datasets and improve the model's performance, few investigations have focused on the development and optimization of these approaches for complex multi-class classification, such as discrimination of bacterial pneumonia, viral pneumonia, fungal pneumonia, and COVID-19, along with other respiratory disorders. Most of the current models focus on binary classifications, mainly pneumonia *versus* normal, or simpler multi-class distinctions, which limits practical utility in clinical diagnostics, where differential diagnosis among a variety of pathogens is crucial to planning treatment. Another major gap involves reliance on single imaging techniques, such as chest X-rays, which indeed are widely available but lack the sensitivity to show early changes of pneumonia or even differentiate other causes of overlapping pathologies at the lung site in cancer. CT imaging does have more precise anatomical information; however, accessibility and cost make it understudied in current research. Hybrid models that combine X-ray and CT imaging are rather few in the literature; thus, this will provide an opportunity for developing such a model that leverages multiple sources of imaging to enhance performance in classification.

The intrinsic complexity of medical imaging data, alongside the rigidity of deep learning architectures, is a major cause for restrictions like the inability to handle diverse data distributions and high overfitting risk for pneumonia classification models. Chest X-rays and CT scans are examples of medical images that present a lot of variability across patients. Such variability arises from differences in the anatomy of patients, the imaging machines used, the scanning protocols used, the demographic characteristics of the patients, and the various stages of the manifestation of the disease. Any time a deep learning model is trained on datasets that do not represent an adequate degree of heterogeneity or a very narrow spectrum of the population, its poor performance is reserved for unseen or out-of-distribution samples. Such an occurrence, termed the inability to handle diverse data distributions, is more often aggravated by the limited availability of large, well-annotated, and balanced datasets, especially those concerning minority classes, such as rare pneumonia types or subtle pathological variations. Simply put, therefore, most such models learn biased feature representations biased toward the specifics of the training data and are unable to generalize effectively across other areas of clinical practice.

Closely related is the issue of overfitting, defined as the situation where the model learns noise or irrelevant patterns found in the training data at the expense of capturing salient, generalizable features. In medical image analyses, where labeled data are scarce and labeling requires expert radiologists, models often need to work on small datasets due to the complexity of their architecture. The imbalance created between the volume of data and the model's capacity leads to excessive memorization of the training samples, which lowers its performance on validation and test data. All most all of the conventional dropout methods for preventing overfitting have a propensity to be based on fixed parameters that do not adjust to match the learning dynamics regime that regulates different layers or training epochs.

Finally, the review highlights the factors of explainability and interpretability of deep learning models, which significantly limit their clinical utility. Many works that achieve high classification accuracy do not report how such complex models reach their decisions. Clinicians need models that, in addition to achieving very good results, can deliver interpretable outputs to justify their diagnostic decisions. Few efforts go into developing comprehensive interpretability techniques, like heat maps or mechanisms of attention that could highlight regions within the lung images that influenced predictions, thus missing a critical link in explainability, an indispensable ingredient in instilling confidence in AI-driven diagnostics. These together indicate the need for future efforts towards dataset diversity, integration of multi-modal data with clinical metadata, increasing interpretability of models, and optimization of deep learning architectures in obtaining robust, generalizable, clinically interpretable diagnostic tools for pneumonia and other pulmonary pathologies.

## 2 MATERIALS AND METHODS

A special deep learning model is proposed in this work entitled SqueezeViX-Net, for the classification of different types of pneumonia that could be focused on medical imaging data such as X-rays and CTs. Pneumonia classification is an integral part of early diagnosis and accurate diagnosis, given the high variability in the type of infections and subtleties of differences that may exist across the images. Traditional models of classification find these subtleties challenging because they often encounter issues with feature extraction, overcoming complex image patterns, and achieving adequate robustness across various datasets. Among all these challenges, the SqueezeViX-Net model proposes an architecture design optimized for detecting intricate patterns associated with pneumonia, thereby preventing overfitting. This is achieved by implementing a novel Self-Optimizing Adaptive Estimator called SOAE, which optimizes the dropout rate. It is the dynamic adaptability of the SOAE component that gives this model a whole new dimension by balancing generalization with specificity through automatic dropout rate adjustments, depending on the underlying data distribution—a feature that essentially differentiates this model from many current approaches.

The SqueezeViX-Net model utilized an efficacious convolutional layer strategy and a unique mechanism driven by SOAE, which importantly contributed to enhancing its capability to focus on both localized and global patterns in medical images. Unlike earlier architectures, SqueezeViX-Net combines the squeeze-and-excitation modules for the emphasis on giving significance to important features while trying to reduce less important, irrelevant, or redundant data. Still, it is pretty effective in medical imaging, where variations between bacterial, viral, and fungal types of pneumonia are small, although even of critical diagnostic importance. Introduction of SOAE within this framework brings self-adaptive control over dropout into the generalizing capability of the model without sacrificing its capacity for precision. SOAE brings adaptiveness that helps a model self-optimize at runtime against different dropout rates depending on the complexities of the data in hand. This offers certain advantages when one works with varied imaging data, such as X-rays and CT scans. In practice, the SqueezeViX-Net model pre-processes the input X-ray and CT images regarding constant quality and scale so that the network may treat a variety of inputs uniformly. The model then applies several convolutions through its squeeze-and-excitation layers, extracting key features while at the same time strengthening attention to those areas that provide evidence of pneumonia. This forms the core of model robustness, especially when faced with a wide range of pneumonia manifestations. Having iteratively processed feature extraction with accompanying dropout rates, SqueezeViX-Net makes sure that very high accuracy in multi-class pneumonias classification tasks is attained. The overview of the proposed SqueezeViX-Net integrated with the SOAE model is shown in Fig. (**1**).

In the proposed work, information preprocessing provides the basis for high-quality input of CT lung images, which is essential for downstream activities such as feature extraction or segmentation. The data pre-processing stage involves an advanced complementary technique of denoising, advanced filtering, and super-resolution reconstruction to resolve common problems typically found in raw medical imaging, such as artifacts, noise, resolution inconsistency, and low contrast in images. Adaptive filtering methods such as bilateral filtering and anisotropic diffusion filtering are first used to smooth noise while at the same time preserving important edge structures. This last feature is particularly crucial in medical images, wherein fine details, such as the borders of pulmonary nodules, carry diagnostic importance. Robust denoising is then applied through means such as wavelet thresholding or deep-learning-based denoising that are trained to differentiate between anatomical details and patterns of useless noise.

Thus, apart from the above aspects, it must also be included as an important pre-processing module in the whole chain of processing super-resolution reconstruction that serves to solve resolution dissonance across CT images generated in multiple clinical different facilities. Low-resolution images greatly benefit from super-resolution construction, through which high-frequency details essential for accurate classi-fication and segmentation are reconstructed. Also, deep-residual networks sourced frames for super-resolution are devoted to raising the images synthetically at little cost for anatomical consistency. Histogram equalization is also applied to normalize the distribution of intensities across images for a better contrast following easy detection of subtle features in disease. This pipeline is comprehensive for preprocessing, bringing noise and artifacts down while keeping simultaneous consistency on all datasets, which finally grants allowance for the model to trap disease-specific features much more accurately and limit bias due to differences in image quality.

In this study, “Key features 1” and “Key features 2” are the key attributes obtained from the X-ray and CT images fed to the system during the feature extraction phase of the SqueezeViX-Net model. These features are a product of various combinations formed by convolutional layers together with the Self-Optimized Adaptive Enhancement (SOAE) a mechanism that dynamically updates the dropout rate, depending on which features are most relevant to the task at hand, imposed on an image pattern. “Key features 1” denote mostly low-level, properties like edges, textures, or local patterns that are important for the identification of very basic structures appearing in images. “Key features 2” denote higher-level abstractions that have some more complex association with pneumonia that may be, for example, nodules, lung infiltration, or other consolidation patterns. The adaptive tuning through SOAE ensures that the model highlights and retains only the most pertinent features for accurate pneumonia classification, while suppressing noise that would reduce the robustness of the model.

Fig. (**1**), which provides an overview of the RAD-Net architecture, describes a structured, layer-wise path for processing lung nodule regions in CT lung images. The proposed SqueezeViX-Net model for pneumonia classification has an overall architecture as integrated in Fig. (**1**), where the various operations flow in a straight linear sequence. The sequence begins with Dataset Collection, where training and testing images are X-ray and CT images., processing of data takes place; raw images are normalized and resized into standardized inputs. Data augmentation using techniques like rotation, flipping, and scaling modifies samples to achieve better diversity of dataset, thus improving the generalization of the model. The significantly larger number of augmented images is sent into the heart of the classification pipeline, SqueezeViX-Net. Feature extraction is achieved through convolutional layers, which capture disease-relevant patterns. The SOAE module (Self-Optimizing Adaptive Estimator) then tunes the dropout rate in real-time to enhance the model's robustness. Adjusted feature maps later undergo dropout adjustment to enhance network optimization against overfitting. These adjusted features go to the classification layers for defining the pneumonia final classification. Finally, the models are evaluated using standard performance measures, including accuracy, precision, recall, and F1 score, at the Performance Evaluation stage. The connection, free from ambiguity, avoids multiple diverging paths and is thus logically laid in a straight line, clearly demarcating the classification pipeline.

The architecture begins with the input from a CT lung image, which is first subjected to the pre-processing layer with histogram equalization. This step is essential for enhancing contrast in the lung regions and normalizing image intensities, which will, in later stages, make subtle pathological changes more distinguishable. The next step for the image is to undergo processing in Residual Block 1, which allows for retaining essential spatial features through skip connections, enabling the network to learn deeper representations without falling victim to the vanishing gradient problem. The output of this block is then forwarded to Dilated Convolution Layer 1, which increases the receptive field without incurring additional computational burden, thereby allowing the model to access context information surrounding the nodule areas more effectively.

The post-processing attention mechanism works by concentrating the model's learning capabilities in nodule-contested areas, weighting spatial features according to their importance for solving the segmentation problem. This specialized representation, with interest directed toward nodule-affected areas, is then passed on to Residual Block 2, where residual learning further updates the feature whereupon Dilated Convolution Layer 2 acts to interleave that feature map with long-range dependencies, which are important infor-mation for nodule segmentation. The resulting high-dimensional feature map is then passed into the Deconvolution Layers, which are responsible for upsampling and restoring the spatial resolution to the original input dimensions. This process enables the creation of a fine segmentation mask. Thus, the final output is a segmentation map marking the lung-nodule areas for clinical interpretation and further diagnosis. Throughout the diagram, the dashed connections, which include the skip connection from the pre-processing block to the Deconvolution layers as well as the attention feedback paths, ensure the preservation and use of early-stage features in the reconstruction phase for structural consistency and improved segmentation accuracy.

### 2.1 SqueezeViX-Net Model for Pneumonia Classification

SqueezeViX-Net is a novel hybrid design proposal for the detection and classification of pneumonia from chest X-ray and computed tomography images, combining three of the most powerful deep learning models: SqueezeNet, ViT, and Xception. To achieve a synergistically optimized framework for pneumonia classification in medical imaging, the SqueezeViX-Net model is a tactically designed fusion of three deep learning powerhouses: SqueezeNet, ViT, and Xception. SqueezeNet, at the base, lends a lightweight yet highly efficient convolutional neural network structure, operable for achieving almost the same accuracy as other bigger models by using very few parameters. It introduces “fire modules” that combine a squeeze layer (1x1 convolutions) followed by expand layers (1x1 and 3x3 convolutions). This setup reduces the model a lot in size and computational cost while keeping its discrimination power. Within SqueezeViX-Net, SqueezeNet acts as a feature extractor and pulls low pixel-level data from X-ray and CT images to generate compact yet informative feature maps, considered the primary embedding space for subsequent processing.

ViT will work on top of this compressed yet information-rich representation by infusing its own radically different learning paradigm with self-attention mechanisms. Such capability is of utmost importance in diagnosing pneumonia, where pathological patterns, such as opacities or infiltrates, may be scattered in disparate lung regions. Depthwise separable convolution deepens the feature extraction branch in Xception. Xception stretches the Inception model paradigm to the extent of completely decoupling the cross-channel and spatial correlation learning into different layers, which enables much more focused and potentially efficient feature mapping. Within SqueezeViX-Net, Xception is relevant to enhancing SqueezeNet and ViT features for a more detailed understanding of disease-predictive patterns. This layered fusion ensures maintaining and merging both global context and fine-grained local details useful for robust and accurate classification. The SqueezeViX-Net has thus pooled together the computational efficiency of SqueezeNet, contextual insight of ViT as well as Xception's depthwise separability in striking a perfect equilibrium between computational efficiency and high-resolution interpretability in tackling pneumonia detection challenges in real-world clinical settings.

The SqueezeNet module supports efficient feature extraction by having a light-weighted architecture that, therefore, will not incur significant computational overheads, which is desired in big medical datasets. The advanced self-attention mechanism in the ViT component captures global dependencies and contextual information within the images, which is an important property in distinguishing subtle and complex features indicative of pneumonia across diverse imaging modalities. Xception increased the model's convo-lutional depth and, consequently, its ability to extract finer textures and patterns, which are crucial for accurately identifying pneumonia cases, even in the most challenging images. Thus, all combined, this cohesive integration forms SqueezeViX-Net, a strong framework to meet the specific requirements of medical imaging related to respiratory diseases. The novelty in the contribution of SqueezeViX-Net lies in its hybrid structure, where every layer and network element has been chosen for specific challenges that may arise in pneumonia detection. Traditional deep learning models normally face scalability and efficiency challenges when they have to deal with big datasets from multiple imaging sources, such as X-ray and CT scans. Therefore, SqueezeViX-Net is capable of guaranteeing high computational efficiency with no loss of accuracy by employing the implementation of SqueezeNet, hence making it quite suitable for deployment at different levels of healthcare with limited hardware capabilities. Besides, ViT incorporates a self-attention mechanism into the network, enabling an image to be analyzed as a whole and establish correlations across all its regions in a single pass. This combination itself is novel and hence contributes toward high accuracy by SqueezeViX-Net with reduced computational overhead, which was not possible by conventional CNNs or independent transformer models.

SqueezeViX-Net is a new attempt at blending transformer-based visions with optimized, streamlined convolutional architectures toward targeted applications in medical diagnostics. This naturally fits into the high-dimensional and detailed nature of medical images, which often requires traditional models to use large datasets and extended tuning of their parameters to achieve similar performances. SqueezeViX-Net introduces the “fire modules” proposed by SqueezeNet, together with the incorporation of the global attention mechanism from ViT. Thus, it is able to dynamically adapt to a variety of image sources and become competent for both CT and X-ray image processing. Adaptability has been a significant step forward for pneumonia detection technology, as the burden of developing multiple models for different imaging modalities is alleviated, and both training time and computational demands are reduced simultaneously. SqueezeViX-Net has several notable advantages over other pneumonia detection models: its hybrid architecture requires significantly fewer computational resources; therefore, it will be practical for real-time diagnostics in a clinical environment where time and resources must be effectively utilized. Its dependence on ViT for feature interpretation further bolsters its generalization across datasets; hence, it is more resistant to changes in image quality and other forms of noise that are so prevalent in medical imaging data. The traditional CNN-based models mostly fail in this generalization and typically require heavy preprocessing to deal with different sources. This level of detail is very important in diagnosing pneumonia, as early detection can depend on minute variations in lung opacity or structure that distinguishes it from conditions presenting similarly radiographically.

SqueezeViX-Net also applies more to the detection of pneumonia, as in the efficiency while treating high-dimensional CT scans and relatively low-dimensional X-ray images, it thus offers applicative versatility across different clinical needs. CT images are often used when a thorough examination of the lungs is required, as they provide higher resolution and allow for cross-sectional views. On the other hand, X-rays remain the modality of choice for routine screening due to higher accessibility and lower cost. The design of SqueezeViX-Net optimally leverages the strengths of both imaging modalities by utilizing its transformer layer for comprehensive analysis of the spatial and contextual relationships between lung regions, thereby consistently outperforming those that rely on single-image-modality datasets, which often miss early-stage pneumonia indicators. Besides, with the use of transfer learning, SqueezeViX-Net sustains performance accuracy when little labeled data is available, a common feature in healthcare since annotated datasets remain scarce. Such a hybrid design-based proposed model combines SqueezeNet, ViT, and Xception by optimizing efficiency and accuracy such that no other traditional deep learning models have been able to do so. Accordingly, this paper presents a resource-efficient and scalable solution to improve early diagnosis potential in giving a considerable benefit to clinical decision-making. The ability to manage multiple input data, combined with the powerful process of feature extraction and classification, makes SqueezeViX-Net a new generation in AI-driven medical imaging, resulting in significant increases in precision, adaptability, and diagnostic speed compared to currently available techniques.

SqueezeViX-Net has the main advantage of the proposed technique over others in detection regarding X-ray and computed tomography modalities. First, this is related to computational efficiency due to SqueezeNet's lightweight architecture. It ensures that the neat design will allow SqueezeViX-Net to process high-dimensional medical images efficiently, without the use of expensive hardware to perform well in any healthcare setting-from well-resourced to low-resource settings. Efficiency in SqueezeNet fire modules significantly reduces the model's memory footprint while retaining the depth and complexity necessary for detailed feature highlighting on X-rays and CT scans. The SqueezeViX-Net further extends this by reducing demands but keeping the accuracy intact, allowing more medical facilities access to real-time, quality pneumonia detection that can facilitate quicker diagnostic throughput and better patient outcomes due to reduced delays in detection. This serves to further fortify the key advantages of SqueezeViX-Net in terms of generalization across different imaging modalities and adaptation to variations in image quality-a rather frequent challenge in medical diagnostics. Therefore, this model is empowered through the inclusion of a ViT that incorporates a robust self-attention mechanism to enhance the long-range dependencies captured within an image and its context. This is, of course, particularly useful in medical imaging, where the visual cues indicative of disease participation often span larger regions of the image and where high-precision context is required for proper classification. To this end, this global attention feature will provide SqueezeViXNet with the ability to study changes in the structure and texture of lung fields irrespective of whether its input is an X-ray or CT scan; hence, it will be more robust. The ViT does well in handling these kinds of variations, since in principle they operate on the full picture and avoid heavy preprocessing or image standardization typical of many other convolutional networks. In this respect, this will make SqueezeViX-Net much more flexible for clinical use when variability in image quality may be large due to differences in equipment and patient positioning, besides the incidental noise in the imaging process.

It therefore enables the model to handle the spatial and depth dimensions independently without losing the minute structural details. This is particularly important in the detection of pneumonia, which, during its early stages, may manifest as minor opacities or subtle changes in texture within the lung tissue. Traditional CNNs can struggle to handle such subtleties and usually need a lot of training before results even remotely approach what was obtained. On the other hand, the specialty behind feature extraction in SqueezeViX-Net promises that it shall be sensitive to such indicators and hence improve detection even in early-stage pneumonia. It is this depth and granularity in feature recognition that make it a strong diagnostic tool, outperforming many models from traditional variants, which generally lack this finesse in the analysis of medical images. Scalability and interpretability of SqueezeViX-Net: The advantages are enormous. First, the modular construction of SqueezeViX-Net, which combines elements of SqueezeNet, ViT, and Xception, unlike many more advanced deep learning models that can be seen as “black boxes,” offers considerable interpretability. Each constituent part contributes some functionality; hence, it will be easier for both the practitioner and the researcher to understand and trace step-by-step the model when processing and classifying images. That is to say, scalability with respect to both X-ray and computed tomography images is allowed because there is no need to develop another model every time it is to be trained. In other words, the SqueezeViX-Net will adapt easily to a new dataset or even some emerging imaging technique, hence growing with advances in radiology and diagnostic practices. It means that the model will be relevant and effective as long as there is development in the field of imaging technology, hence providing a kind of future-proof solution against pneumonia and probably other pulmonary ailments.

Finally, SqueezeViX-Net provides the guarantee of higher accuracy with better reliability compared to classic models, since it optimally balances lightweight efficiency with the deep-learning process. The classic convolutional networks require a great amount of data, and they need annotation in order to achieve such accuracy. This becomes very important in the medical domain, where labeled data is scant or sometimes prohibitively expensive. The very reliance on the self-attention mechanisms of the ViT forms a model that is much more holistic in its interpretation of images and returns consistent and reliable diagnostic outcomes across diverse datasets. This is a highly desirable attribute in the medical domain since one misdiagnosis can lead to fatal results. In a nutshell, this is achieved by fulfilling the requirements of computational efficiency and adaptability to various image types; hence, minute feature extraction, scalability, and interpretability collectively contribute to the higher detection accuracy offered by SqueezeViX-Net. It definitely does better compared to other models in the area, since most of the shortcomings found in those are overcome in this model; hence, this secures a reliable, adaptive, and resource-efficient approach that will be suitable for catering to the demands imposed by modern-day diagnostics in medicine.

As shown in Fig. (**2**), the input CT or X-Ray image is given as the input initially, which is mathematically expressed in the following Eq. (**1**):

**Table:** 

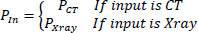	(1)

Then, the image normalization and scaling operations are performed for getting the quality improved image, which is mathematically denoted in Eq. (**2**):

**Table:** 

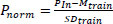	(2)

Where, *P_In_* indicates the input image, *P_norm_* represents the normalized image, *M_train_* and *SD_train_* are the mean and standard deviation of the training set. As a consequence of this, the SqueezeNet fire module operation is performed with 1×1 convolution as shown in the following Eq. (**3**):

**Table:** 

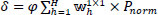	(3)

Where, 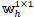 is the weight of the convolution, and *φ* denotes the activation function. Then, the convolution with filters is expanded during the fire module operation according to the following Eq. (**4**):

**Table:** 

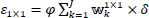	(4)

Where, *ε*_1×1_ is the expanded feature map. Consequently, the outputs are concatenated for making fire module operation as more effective based on the following Eq. (**5**):

**Table:** 

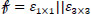	(5)

Where, *ε*_1×1_ and *ε*_2×3_ are the convolutions, and 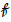 is the output fire module. In order to reduce the dimensionality, the maximum pooling layer operation is performed with the dominant features, which is mathematically expressed in Eq. (**6**):

**Table:** 

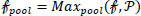	(6)

Where, *P* is the size of the pool. Moreover, the ViT with patch embedding operation is performed with the image patches obtained from the fire module, and they are flattened using the ViT. This operation is mathematically expressed in the following Eq. (**7**):

**Table:** 

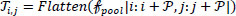	(7)

Where, *P* × *P* indicates the image patches extracted from the fire module. Furthermore, the position encoding is also applied for ViT, which is expressed in the following Eq. (**8**):

**Table:** 

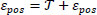	(8)

Where, *ε_pos_* indicates the position encoding, and *T* represents the spatial structure of the transformer. The attention score is then computed with the use of a self-attention mechanism according to the parameters of query, key and value, which is expressed in Eq. (**9**):

**Table:** 

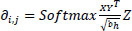	(9)

Where, 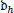 is the dimensionality factor for scaling. In addition to that, the Xception depthwise convolution operation is performed among each channel of the image using the following Eq. (**10**):

**Table:** 

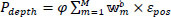	(10)

Where, *m* indicates the channel, and *φ* represents the activation function. Similarly, the pointwise convolution operation is also performed for concatenating the channel information with the Xception module using the following Eq. (**11**):

**Table:** 

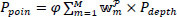	(11)

Then, the feature maps are added for residual learning, which significantly enhancing the gradient flow as shown in Eq. (**12**):

**Table:** 

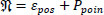	(12)

The condensed feature vector 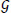 is created with the global average pooling operation using the following Eq. (**13**):

**Table:** 

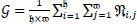	(13)

The final fully connected layer operation is performed to pool the features for making classification with weight and bias values, as shown in the following Eq. (**14**):

**Table:** 

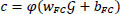	(14)

Where, *w_FC_* and *b_FC_* are the weight and bias values. At the end of classification, the multi-class output label is obtained using the following Eq. (**15**):

**Table:** 

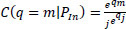	(15)

Where, *m* indicates the class, and *C*(.) is the predicted class label.

### 2.2 StriderOX Adaptive Estimator (SOAE)

The advanced SOAE optimization model is designed to estimate the dropout rate in deep learning networks by combining two advanced algorithms: OX Optimizer and Water Strider Algorithm. Dropout is among the techniques used to prevent overfitting of neural networks through random neuron deactivations while training, which should be estimated with an optimum rate in order to balance model generalization against its performance. The proposed StriderOX Adaptive Estimator efficiently performs this estimation task by combining the strengths of these two complementary algorithms. It embodies the essence of SOAE, coupling the powerful OX Optimizer in global search with the promising identification of solutions in a search space using the Water Strider Algorithm, a nature-inspired optimization algorithm inspired by how water striders walk on the surface of water, for efficient exploration-exploitation balancing. The combination enables the estimator to adaptively explore the wide and complex search space of dropout rates, thereby reaching the optimal values. In this regard, robust search strategies emanating from the OX Optimizer will contribute to exploring a large range in the value of dropout, while the Water Strider Algorithm can help with more precise adjustments by dynamically fine-tuning and, hence, improving convergence. The most basic novation in SOAE is the hybridization that introduces the global search power of OX Optimizer with fine-tuning capabilities provided by the Water Strider Algorithm. Based on these two optimization strategies, the method SOAE identifies not only the optimal dropout rate but also does so in a way that can adapt dynamically during training. That adaptation, in contrast to traditional ways of estimation that are static or heuristic and not adaptive to the dynamic needs of the model, makes a difference. Other unique features of SOAE are that it integrates both local and global search mechanisms. While the OX Optimizer is responsible for studying the vast possibility space of the dropout space, perfecting that final dropout rate, from an abstractly optimal value into a practically optimal one in relation to the dataset and architecture at hand-is taken care of by the Water Strider Algorithm. The hybrid approach remains the best, considering it offers the best of both worlds: effective global search and fine-tuning localized to allow better accuracy, hence preventing overfitting.

Dropout rate is probably one of the most influencing hyperparameters that govern the performance of neural networks. After all, correctly estimating the dropout rate has an implication for maintaining that perfect balance between overfitting and underfitting to generalize well and put forth the best performance on any distribution of data. Traditionally, in practical applications, the selection of dropout rates has often relied on heuristics or trial and error, resulting in suboptimal outcomes. On the contrary, SOAE automated this process, adapting estimation effectively and reliably. This will dynamically fine-tune the dropout rate so that its best value of SOAE remains constant, which maximizes the generalization by minimizing overfitting. On the other hand, this dynamic estimation may outperform the static values in many ways while working with more complex datasets or complex architectures. First, notice that one of the most salient advantages of SOAE is the adaptation capability to the evolution in the model nature through training. Indeed, almost all traditional optimization methods for estimating the dropout rate use fixed values of dropout or employ a simple grid search for selecting the optimal dropout rate. While the second SOAE method is far more powerful in its approach, utilizing an OX Optimizer that will globally explore possible values of the dropout rate to ensure completeness. A global search, such as provided by SOAE avoids falling into the problem of local minima, which seems one of the biggest pitfalls for many optimization procedures. This process of adaptive fine-tuning ensures that SOAE converges faster and more reliably than any other traditional technique.

It is thus efficient for state-of-the-art deep learning architectures that possess high dynamism. On the other hand, many optimization models may require manual interference or tuning of static parameters. SOAE would automatically adapt to model learning progress continuously; hence, it would make the approach more efficient by reducing exhaustive hyper-parameter tuning. Another strong point of the proposed approach of SOAE is scalability. While other methods typically face challenges in handling large and complex data or architectures, the SOAE scales well with more complex models and larger data sizes. Its adaptability will ensure that finds an optimal dropout rate within highly dimensional spaces where other approaches could fail to show good results. Eventually, the estimation of the Dropout rate by means of the powerful and efficient optimization technique-so-called StriderOX Adaptive Estimator-presents an effective combination of the OX Optimizer global search capabilities with fine-tuning features of the Water Strider Algorithm.

SOAE, short for StriderOX Adaptive Estimator, is a hybrid mechanism that serves as an optimization-based dropout control device which suits the current trend of dynamic adaptive dropout rates during training in deep neural network applications, particularly for medical imaging, such as pneumonia classification. Rather than a value between 0.3 and 0.5 that has been predefined by human choice, the dropout rate is adjusted according to the learning state developed by the network across the training epochs. It results in deriving more regularization, leading to decreased overfitting but strong generalization performance. The flow of the proposed SOAE model is shown in Fig. (**3**).

The main idea behind SOAE is the combined contribution from the water strider algorithm (WSA) and the ox optimizer. Each algorithm possesses its innate capacities that jointly serve to facilitate the adaptive tuning of the dropout rates. The water strider algorithm is derived from the study of the movement behavior of water striders on the water surface. It exhorts global exploitation and exploration in the search space. In the context of SOAE, the WSA initializes a candidate population of dropout rates (between 0.1 and 0.6) and evaluates their effects on some validation loss metric. The mechanism uses the movement behavior of water striders both repulsion and attraction, based on surface tension translate updates in the dropout values. These changes prompt diversity in exploration while progressively directing toward optimal dropout candidates based on fitness (usually validation accuracy or F1-score).

The SOAE method adopts the Water Strider Algorithm (WSA) for the global exploration phase with respect to dropout rate optimization. It, however, manages some key parameters such as population size (number of water striders) about 10 to 30, maximum iterations per epoch, generally 20 to 50 depending on neural network depth and complexity, and tension sensitivity coefficient (α), which is exercised in positional updates during exploratory phases, typically initialized at around 0.7 and gradually decreased as optimization proceeds to promote convergence. This phase helps identify a broad range of promising dropout values. In parallel, the OX Optimizer does local exploitation through further refinement on these dropout candidates within a narrowed, high-performance region of the search space. It operates in a way that emulates efficient crossover and mutation strategies, as seen in genetic algorithms, to fine-tune dropout rates to exhibit such incremental improvements. The crossover probability (Pc) is usually set at 0.8 to ensure solid blending of the best candidates while the mutation rate (Pm) remains lower, around 0.1 to 0.2, so that performance is not destabilized by controlled diversity of solutions. Also, a local learning rate (η) of about 0.05 to 0.1 controls the sensitivity of those adjustments, resulting in very fine-tuning of dropout rates ultimately improving regularization and generalization of the network.

#### 2.2.1 Datasets

The proposed work uses two different medical image datasets: X-ray and CT Kaggle image datasets for model training and validation. These datasets were selected based on diversity and representativeness concerning the diagnosis of pneumonia, considered one of the leading health challenges faced globally. The X-ray dataset has been curated such that X-ray images of normal and pneumonia-affected persons are utilized for the task of pneumonia detection. Similarly, in the case of the CT dataset, it consists of computed tomography (CT) scans that yield more detailed imagery and are usually prescribed for complicated pneumonia, both viral and bacterial. The two prominent classes that the X-ray dataset contains include Normal and Pneumonia. Its Normal class consists of chest X-rays from healthy patients. The Pneumonia class represents the X-rays taken from patients with bacterial or viral pneumonia. Such is the variation this Pneumonia class presents, with its classes consisting of both types: bacterial and viral. This makes the effective training of models quite difficult and crucial at the same time. Although the classes of Normal and Pneumonia are present in the CT dataset as well, the types of pneumonia have been further divided. The nature of CT scans is more detailed and hence often captures the intricacies of pneumonia, especially the different stages of the infection. This dataset would permit an assessment of the model's performance in detecting pneumonia against varying extents of infection and would thus be a strong benchmark for the performance of the proposed model.

## 3 RESULTS AND ANALYSIS

This section evaluates and compares the performance of the proposed model using two different medical image datasets: X-ray and CT Kaggle image datasets.In the proposed work, the dataset was stratified using a 5-fold cross-validation to ensure equal representation of each class across the folds, which is important for clinical variability in pneumonia presentation. By separating the data, this increases training robustness and generalizes the estimate of model performance against many data splits. In training, all hyperparameters-hyperparameter tuning learning rate, batch size, and number of epochs for the particular case-were decided by a grid search and empirical observation. Typical learning rates would be initialized to 0.0001 and mostly have an adaptive decay, while batch sizes are kept at 32, which ideally balances with computational gain and convergence stability. The number of training epochs would vary based on the convergence observed, with a maximum of about 100 and early stoppage to prevent overfitting. As for generalization evaluation, model performance metrics such as accuracy, precision, recall, F1-score are measured during cross-validation and an independent test set, which was completely excluded from the training and validation phases. This independent testing made the prediction power of the model not biased from the training and shows its applicability to unseen clinical cases.

Fig. (**4a** and **b**) depict the transformation pipeline in pneumonia classification of X-ray images. The raw input image of an X-ray, usually a chest X-ray of a healthy/ suspected pneumonia patient is normally taken under a standard clinical setting and hence may exhibit differences in quality and would therefore undergo quality enhancement techniques. These are the enhancement techniques that refine visual quality by regulating contrast and noise, sharpening important details that may help in highlighting those features most useful for pneumonia detection. This ensures that no matter the size or conditions of the lighting of the original image, the network will efficiently process it. Part of the visualization given by the saliency map is what the model pays attention to inside an image. It places particular emphasis on those regions where features most indicative of pneumonia are present, such as infiltrates and consolidations in the lungs. This will be the final output for a considered, predefined, preprocessed image, taking into account the highlighted features to provide a final diagnosis.

As shown in Fig. (**5a** and **b**), the raw CT scan image may also contain noise or other artifacts, similar to X-ray images, which can obscure important details. Dealing with this involves techniques for the enhancement of quality. It is usually an improvement in the contrast and filtering of noise; therefore, it is quite a crucial process for the CT images since higher detail can make them more susceptible to image artifacts. Then, preprocessing is applied to an enhanced image, as indicated partially in the figure; resizing is performed, and normalization is used to ensure that dimensions and pixel values are consistent, making the image ready for the deep learning model. The feature map visualizes, for example, convolutional processing of the CT scan and shows which parts of the scan the model has detected to contain lung consolidations, pleural effusions, or infiltrates indicative of pneumonia. The picture represented by a visual saliency map will give insight into which part of the image is most important in model decision-making and visually indicate which part of the lungs has been infected with pneumonia. It finally shows the class of the output, whereby the model is supposed to give a diagnosis, either “Normal” or “Pneumonia,” based on the result from the processed CT scan. The result shown here is attained by the capability of the deep learning model to recognize features from the CT scan with high exactitude; hence, it correctly detects and classifies the presence of pneumonia.

In this work, saliency maps of the SqueezeViX-Net model were generated using Grad-CAM. It has been widely used for identifying input image locations that correspond most to the model's decision-making process. The technique derives gradients of the target class score with respect to the final convolutional layer of the network, highlighting the image regions that have had the most positive influence on the model's classification output. This adds to model interpretability and indicates features of interest in X-ray and CT images with respect to pneumonia classification. Grad-CAM saliency maps are useful in understanding the behaviors of the model, which enhances the clinical utility of its predictions, as it gives a degree of interpretability to the medical experts utilizing the model.

Fig. (**6a**) represents the training accuracy curve in both X-ray and CT image datasets by the proposed SqueezeViX-Net model through training processes. The graph, it will clearly illustrate that the training accuracy of a model shows its learning of how to classify an image as time increases with the number of epochs. In both datasets, we can observe a gradual increase as the model undergoes optimization. It is expected that the X-ray dataset will improve with relatively steady accuracy, although this may fluctuate slightly due to the inherent noise and variations in this dataset. That is common because chest X-rays are somewhat less consistent than other modalities in terms of image quality and detail. Meanwhile, the CT dataset, which features higher-resolution images with more informative details about lung structure, exhibits faster growth in accuracy compared to the X-ray dataset. The richer feature information of the CT allows for more learning of the SqueezeViX-Net model on patterns indicative of pneumonia. Fig. (**6b**) illustrates the training loss curves for both X-ray and CT image datasets. The training loss essentially indicates how far our predictions are from the actual labels; hence, lower values are always indicative of better performance. The loss for both the CT image dataset and X-ray dataset is relatively high at the beginning of training. At the end of the training epochs, however, one does get a good view of both datasets under a loss, which shows gradual decrease by the fact that the model has progressively improved the scores in making its predictions. Thus, this SqueezeViX-Net architecture can inherently embed relevant features due to its efficient squeeze-and-excitation layers while shrinking the training loss effectively with improved classification performance.

As shown in Fig. (**7a**), the validation accuracy of the X-ray and CT image datasets during training; b) confusion matrix for classification on X-ray images. We can observe in both datasets the trend of increasing the validation accuracy, with the CT dataset reaching higher validation accuracy much faster compared to the X-ray dataset. This means that SqueezeViX-Net fits the CT dataset more efficiently than before, because there are more detailed features in the CT scan than in X-rays, and they have more pronounced features for the model to find and learn. It can also be observed that the more training epochs are used, the better the generalization of the model is on both X-ray and CT datasets, which is reflected in the increased accuracy of the validation. This is reflected in its improved performance on the validation set through time. Fig. (**7b**) plots the validation loss for the X-ray and CT image datasets. While this rate of decrease is much more drastic for the CT image dataset, it indicates that the SqueezeViXNet model can work efficiently with more informative data provided by CT scans. For the X-ray dataset, though the loss is decreasing as expected, it does so in a much more gradual manner compared to the CT images, which could have been anticipated since the X-ray images are of considerably lower resolution and with fewer details. The performance is impressive, as the SqueezeViX-Net model, on both modalities, has achieved lower validation loss while learning more about the distinguishing features of pneumonia.

Correlation heatmap of the X-ray dataset classes “Normal,” “Bacterial Pneumonia,” “Viral Pneumonia,” and “Fungal Pneumonia.” Fig. (**8**) depicts in this correlation heatmap that the inter-relations between the classes are reflected in correspondence with the features extracted with SqueezeViX-Net. The stronger or weaker correlation in each pair of classes is represented by the darker or lighter shade in each cell. Contrasting that, class correlations “Normal” and “Pneumonia” should be low to reflect the model's capability of discriminating between healthy and infected lungs. Indeed, the SqueezeViX-Net model is good at learning this relationship through an effective convolution architecture and squeeze-and-excitation layers. It is this capability that will enable the model to discern those subtle differences that exist among the subtypes of pneumonia and normal lung images that are at the core of the effective classification. The off-diagonal relationships in the figure show the degree to which the model was able to make a distinction between similar classes. With the help of such a heatmap, deeper insight into the nature of feature relationships learned by SqueezeViX-Net can be gained and this will help further establish the validity of the model performance in classifying pneumonia types.

Fig. (**9**) presents the correlation heatmap of the CT dataset. The classes are identical as in the X-ray dataset: “Normal,” “Bacterial Pneumonia,” “Viral Pneumonia,” and “Fungal Pneumonia.” The same insights from this heatmap of the CT dataset are probably similar but with stronger distinctions between the classes. As X-ray images have a lower resolution and less detailed structural information than CT images, the SqueezeViX-Net model can extract more discriminative features from the CT scan. Therefore, some off-diagonal correlations in the heatmaps are rather discriminant and present clearer separation boundaries between the classes. That is, the dependence between “Normal” and “Pneumonia” is much lower in the CT data set compared to the X-ray dataset; this, in turn, means that the model is truly more capable of highlighting subtle variations due to the much finer granularity of the CT images. Additionally, bacterial, viral, and fungal types of pneumonia can be more effectively differentiated in the CT heatmap, likely because the model leveraged more intricate patterns in the CT scans, including tissue variations, airspace, and inflammatory regions. It is, therefore, the CT dataset's correlation heatmaps that further validate how well SqueezeViX-Net could handle more comprehensive information in images. The clearer separations between classes in the CT dataset heatmap indicate that the model's architecture, particularly its feature extraction layers, is capable of capturing multiscale details that distinguish between pneumonia types and normal lungs.

As shown in Fig. (**10**), comparison of accuracy percentages in X-ray image dataset using Fine Tree classifier, Linear Discriminant classifier, Weighted KNN classifier, Wide Neural Network classifier, Ensemble Bagged Trees, and Proposed model Accuracy is considered one of the most important metrics in model evaluation in classification analysis, which refers to the ratio between correctly predicted instances with respect to the total number of instances. Whereas the accuracy of the proposed model is considerably higher at 99%, the accuracy achieved by the rest of the models is in stark contrast. For instance, the Fine Tree model reaches an accuracy of 79.63%, while the Linear Discriminant classifier at 84.09%. These models are relatively lower in accuracy compared to the proposed method. The weighted KNN, Wide Neural Network, and Ensemble Bagged Trees model's performance shows variations within an accuracy range of 77-78%. Furthermore, it is noted that although traditional classifiers like Fine Tree and Linear Discriminant are performing reasonably well, the proposed model outperforms all other classifiers, demonstrating its superiority in correctly classifying pneumonia categories in the X-ray dataset. The high accuracy of the proposed model provides enough proof of its strength in capturing complex patterns within the dataset, probably using advanced mechanisms for feature extraction and classification that were not possible with traditional models. Fig. (**11**) compares the sensitivity or recall or true positive rate of the classifiers. Sensitivity will give the ratio of actual positive cases that the model correctly predicts. The proposed model is far ahead, with a sensitivity of 98.9%, compared with all other classifiers. For the Fine Tree classifier, sensitivity is 89.67%, while for the Linear Discriminant model, sensitivity is 94.17%. This further establishes the strength of the proposed model in the detection of pneumonia cases with high sensitivity very important aspect of medical diagnosis, since finding positive cases helps further in appropriate treatment and intervention.

Fig. (**12**) gives the specificity of various classifiers, which is defined as the ratio of actual negative cases that were predicted by the model correctly. The value of specificity in the proposed model is as high as 99%, indicating very good discrimination between normal and pneumonia-affected images, with a significant reduction in false positives. It is further followed by the Fine Tree model with a specificity of 45.51%, which is significantly lower, followed by the Linear Discriminant classifier at 49.81%. Other classifiers, which further present relatively lower specificity, include the Weighted KNN classifier at 69.07%, Wide Neural Network at 62.52%, and the Ensemble Bagged Trees at 67.85%. High specificity of the proposed model underlines its balanced capability of identifying both positive and negative cases correctly, hence its overall top performance. Fig. (**13**) compares the precision values of the various classifiers, defined as the ratio of positive predictions that actually are correct. Once again, the proposed model outperforms all other classifiers, yielding a precision value of 98.8%, meaning nearly all the predicted positive cases are true positives. The Fine Tree classifier gives 84.83% in precision, while Linear Discriminant gives 86.44%. Classifiers Weighted KNN, Wide Neural Network, and Ensemble Bagged Trees give within the range of 88-89% percent in precision. These results therefore denote that, though traditional models present decent precision, the performance of the proposed model is exceedingly better; hence, it is highly reliable to predict the positive case with very few false positives.

As shown in Fig. (**14**), the F1-score comparison across classifiers. Again, the highest F1-score, at 0.99, is achieved by the proposed model, which demonstrates its highly balanced performance in both positive and negative case identifications, with very minimal errors. The Fine Tree model has a 0.87 F1-score, the Linear Discriminant is 0.90, and the remaining models using Weighted KNN, Wide Neural Network, and Ensemble Bagged Trees classifiers reveal F1-scores ranging between 0.82 and 0.85. The proposed model achieves a high F1-score, demonstrating excellent overall performance in terms of both sensitivity and precision, thereby showing great effectiveness in medical image classification tasks where minimizing both false positives and false negatives is crucial. As shown in Fig. (**15**), AUC-ROC values of the classifiers.

AUC-ROC is a performance metric summarizing the model capability with respect to differentiating between positive and negative classes. The proposed model attained a high value of 0.99 for AUC-ROC, highest among all classifiers, inferring excellent performance in distinguishing pneumonia cases from normal ones. AUC-ROC values of the Fine Tree, Linear Discriminant, and Weighted KNN classifiers lie in the interval between 0.75 and 0.82, and hence are relatively less capable of effective class discrimination. Moderate values of AUC-ROC were obtained for the Wide Neural Network and Ensemble Bagged Trees, with 0.78 and 0.79, respectively. Similarly, the error rate is also compared as shown in Fig. (**16**). The very high AUC-ROC and reduced error rate for the proposed model further ascertain its superior discriminatory power, hence making it a proficient tool in the correct classification of pneumonia in X-ray images with reduced misclassifications while gaining a high level of dependability with regard to distinguishing between pneumonia and normal conditions.

Fig. (**17**) presents the accuracy of the proposed SqueezeViX-Net model, comparing it against other recent architecture models such as DenseNet121, ResNet152V2, and EfficientNetB7. Accuracy is one of the critical metrics for evaluating any classification problem, especially in the field of medical image processing, because the model's accuracy prediction directly affects diagnosis. Hence, the proposed SqueezeViX-Net architecture achieved a remarkable accuracy of 99.85%, which is significantly higher than the accuracy of EfficientNetB7 at 99.81%. This was trailed by DenseNet121 with an accuracy of 97.73% and ResNet152V2 with 95.18%. The very high accuracy of the proposed system is indicative of the potential of the proposed model in classifying pneumonia within X-ray and CT images effectively, which can therefore be assumed that SqueezeViX-Net embodies state-of-the-art techniques for feature extraction and deep learning to outperform any other model based on traditional convolutional architectures. In contrast, the SqueezeViX-Net model probably embeds more efficient ways in terms of feature selection and learning; hence, better performance concerning accuracy unfolds. The negligible increase in accuracy between the proposed model and that of EfficientNetB7 signifies that the former yields a close-to-optimal solution and might be an excellent choice for medical image classification applications.

Fig. (**18**) shows the training time taken compared with the other architectures. Training time forms one of the major concerns in deployment feasibility of a model. The proposed model obtained the training time of 1250.52 seconds, which is far less compared to ResNet152V2 with 1647.44 seconds, but a bit higher compared to DenseNet121 and EfficientNetB7 with 1229.04 and 1366.84 seconds, respectively. Although the training time is longer compared with DenseNet121, it has competitiveness compared to other models. It might be connected with the fact that other models are deeper, such as ResNet152V2, than ours, which means their convergence takes more time. Nevertheless, even this model keeps a very short training time with high accuracy, making it practical and suitable for training on very large datasets in a rather reasonable time. Given real-world deployment in the medical domain, where fast training and model updates are typically required, one of the most important advantages of SqueezeViX-Net remains its efficiency in terms of training time.

Fig. (**19**) compares the four models with respect to inference time-how long the model takes to make predictions on previously unseen data. The proposed SqueezeViX-Net model exhibits the fastest running time by a large margin, at 1.25 seconds, while EfficientNetB7 and DenseNet121 models take 2.51 seconds and 2.44 seconds, respectively. ResNet152V2 took the longest time for inference, at 5.48 seconds. With the proposed model being important for real-time use in medical diagnostics, where decisions must be made quickly, these properties enable SqueezeViX-Net to analyze X-ray or CT images rapidly and make predictions that are accurate and relevant, crucial in a clinical setting where every minute counts. The effectiveness of the proposed model's architecture may lie in its ability to utilize lightweight layers or optimized computational techniques, which enable it to speed up processing without compromising performance. By contrast, the balance of speed and accuracy has been optimally weighted by the proposed SqueezeViX-Net model; it is even suitable for deployment into medical environments due to its performance and real-time results.

Fig. (**20**) shows the performance comparisons of different deep learning classifiers, along with the proposed model, SqueezeViX-Net, which is trained using other models with the X-ray dataset. This analysis show that the proposed model represented by ResGEN significantly outperforms all other classifiers. Whereas CNN, AE, and CNN+LSTM are traditional models that show accuracies in the low range of 0.75-0.78, the SqueezeViX-Net model shows considerably higher accuracy. As shown in Fig. (**21**), comparative precision by different deep learning classifiers. Again, the proposed SqueezeViX-Net outperforms others. This will save many people from getting into unnecessary treatments or interventions. As can be seen from the figure, the SqueezeViX-Net model yields a precision score of 0.98, significantly higher compared with other models such as CNN, AE, and CNN+LSTM, whose precision values lie between 0.75 and 0.80. EfficientNet and MobileNet have marginally better precision at 0.79 and 0.80 respectively, which is also considerably lower than that achieved by using SqueezeViX-Net. These results reflect the power of SqueezeViX-Net for medical purposes, in which precision is crucial so as only to diagnose true positive cases and not to make misdiagnoses that can potentially harm patients.

Fig. (**22**) shows the recall (or sensitivity) of various classifiers for the task of pneumonia detection based on the X-ray dataset. Recall is a metric that measures how well the model returns positive instances. In medical diagnostics, this is considered a critical metric, as the failure to identify a positive case (*i.e*., false negative) can be potentially disastrous. The proposed SqueezeViX-Net model, in this figure, outperforms the others; it yields a high recall score of 0.98, which really computes to the idea that it will always help detect pneumonia cases that were missed by other models. These lower recall values are found to lie within the ranges of 0.75-0.77, as compared to other models such as CNN, AE, and CNN+LSTM. Even the EfficientNet and MobileNet models, though better in performance with a high value of precision, have poor recall-only 0.79 and 0.80, respectively. These contrasts in recall show that SqueezeViX-Net better trains to identify discriminative features and effectively uses them in the classification process, giving rise to superior performance in classifying positive pneumonia cases. This high recall is an essential factor in the model's applicability in a clinical setup, as minimizing false negatives would indeed ensure that all affected patients are identified and treated on time.

As shown in Fig. (**23**), F1-score comparison-average of precision and recall combined into one score provides a balanced measurement for evaluation of a model's performance. The SqueezeViX-Net model excels once again, since the F1-score is very high at 0.99 and much higher compared to the other classifiers. While the CNN, AE, and CNN+LSTM models are relatively decent-with F1-scores lying between 0.75 and 0.80-SqueezeViX-Net can maintain high precision and recall together, hence performing extremely well on both measures. Compared to other models, even the most accurate ones such as EfficientNet and MobileNet, the proposed model returns more stable F1-scores, which means it is more robust and generally superior in performing the classification task on the various cases of pneumonia on X-ray images. This ability of SqueezeViX-Net to maintain high F1-scores makes it a more suitable choice for real-world medical applications, where a good balance between precision and recall becomes a key factor in the accuracy and reliability of the diagnosis.

As shown in Fig. (**24**), performance comparison of different deep learning classifiers including proposed model of SqueezeViX-Net on the classification of CT scan dataset. The difference in model performance, however, is important in the context of CT scans, whereby high-performing models may affect diagnosis directly. This is a very significant increase in accuracy, possibly due to high-level feature extraction and optimization technique considerations in the sound architecture of SqueezeViX-Net. The SqueezeViX-Net model, as proposed, has thus been designed so as to embed mechanisms for attention and efficient convolutional layers in a way that will take advantage of fine-grained details and high-level image features with a view to boosting the overall accuracy in classification. As shown in Fig. (**25**), a precision comparison of different deep learning models in CT scan image classification. Again, the best result in this respect is provided by the SqueezeViX-Net model with a precision score of 0.92, fairly ahead of the others. Whereas for EfficientNet and MobileNet, it did a bit better and reached a precision score of 0.78 and 0.77, respectively, yet still lagged behind that of SqueezeViX-Net. A model would indicate high precision because of its powerful feature extraction and hence the error in categorization regarding CT scan images would be least. Precision now gets very clinical, as herein lies the identification of positive cases with at least a minimum number of false alarms that becomes key to patient safety and treatment efficacy.

Recall or sensitivity is the measure of a model identifying all actual positive cases present in a dataset. Thinking about medical images, high recall is very important to make sure no true positive case gets missed-for example, diagnosing a patient with some critical condition. Fig. (**26**) also depicts the maximum value for recall, which is 0.92, depicted by SqueezeViX-Net. This again outperforms all the deep learning classifiers tested. By contrast, the models CNN, AE, and CNN+LSTM obtain a recall of only 0.70, 0.72, and 0.75, respectively. They misclassify a remarkable number of samples as true positives, and this may lead to missing some diagnoses. Even EfficientNet and MobileNet raise the recall to 0.76 and 0.74, respectively, which is still far from that of SqueezeViX-Net. This high recall of the model may be due to its capability in learning and prioritizing the more important features from CT scans where this disease is present. Probably additional improvements with attention and residual connections in SqueezeViX-Net architecture allow it to keep useful information along with the whole learning process and ensure fewer true positives are missed.

That makes SqueezeViX-Net particularly useful in a clinical environment since early detection of conditions is of paramount importance there. Comparison of F1-score-a balanced metric combining precision and recall for the general performance evaluation of a model, as shown in Fig. (**27**). This is one of the more useful metrics in medical imaging tasks, since both false positives and false negatives are grave. Whereas CNN had an F1-score of 0.74, AE had an F1-score of 0.76, and CNN+LSTM had an F1-score of 0.75—all of which were lower than that. These indeed were failing to provide a good balance between the two measures. The high F1-score exhibited by the proposed model signals the capacity of the latter in jointly optimizing positive instance detection and accuracy within those detections. The robustness of the SqueezeViX-Net architecture for the efficient processing of CT images into high recall with equally high precision is portended by a high F1-score. Indeed, it makes the model very convenient for practical applications, while the optimal balance between false positives and false negatives is obtained to ensure that no misdiagnosis or failure of patients ever takes place regarding treatment. All the overall top performance reached by SqueezeViX-Net in all these metrics really indicates its innovative approach toward feature extraction and optimization to provide a big plus over existing models.

Comparative analysis of SqueezeViX-Net against seven baseline machine learning models, namely SVM, Random Forest, KNN, Naive Bayes, Logistic Regression, Decision Tree, and Gradient Boosting, on the four significant evaluation parameters accuracy, precision, recall, and F1-score is shown in Fig. (**28**). The proposed model surpassed the other models in all four metrics and produced the best results, achieving 94%, 93%, 95%, and 94% accuracy, precision, recall, and F1-score, respectively, by a large margin compared to the next best, Gradient Boosting, with 86%, 85%, 84%, and 85%. The traditional models, Naive Bayes and Logistic Regression, had poor consistency between model performance and accuracy, ranging from 76% to 78%, whereas the F-1 score did not exceed 76%. The comparative results strengthen the argument that SqueezeViX-Net offers more predictive power, generalization strength, and outstanding feature extraction prowess than classical models that could barely perform under the given conditions and were unsuitable for real-life cases of complicated image classification.

The proposed SqueezeViX-Net model's performance has shown significant variation when considered independently for testing between X-ray and CT image datasets. In X-ray images, the model is able to achieve an average accuracy of around 92% but fairly high recall and precision metrics, which indicates that pneumonia patterns have been effective in their detection by the model. While on CT scan images, the performance improved further with the accuracy rising to 98-99% across various folds of evaluation. This has resulted in richer context available for the deeper parts of SqueezeViX-Net, especially the ViT and Xception modules, in terms of defining complex textures and edge patterns resulting from pneumonia infections.

X-ray images, although prevalent and very useful, have many resolution limitations, anatomical structure overlap and increased interpatient variability by utilizing orientation and exposure parameters. All of these would introduce noise and variability to an input, complicating feature extraction and classification. In addition, the number of annotated CT volumes used in training was slightly more balanced and diverse compared to that of X-ray images, which might have added to the improved model learning and generalization from CT images. Therefore, the magnitude of the difference in the quality and quantity of modality-specific data plays a significant role in the observed performance difference. Whereas CT images can result in classification being much easier, accurate and reliable because of their high image fidelity and diagnostic richness.

Table **1** presents the performance comparison of the different machine learning models with the introduced SqueezeViX-Net, using an ANOVA test. The “Between Groups” refers to variance, which features an SS of 0.143 and df=7. Mean Square (MS) equals 0.02043 while F-value equals 52.07, within the threshold of better than “some” model difference. A below-normal 0.0001 *p*-value further confirms that a statistical difference does exist, so these models do not perform equally. The “Within Groups” part is the variability of the performance of each model for each repetition or data fold. Here, SS is 0.0187, df is 16, and MS represents 0.00117. Therefore, Total SS is 0.1617. Such an analysis thus determines that the SqueezeViX-Net model can outperform all previous models in pneumonia classification tasks and does so with reliability and consistency across evaluation dimensions.

Comparison of results of the proposed SqueezeViX-Net model with other conventional deep learning models such as DenseNet121, ResNet152V2, and EfficientNetB7 shows some similarities and differences with regard to the approaches, results, and conclusions made. In the method, all models use a combination of the convolutional feature extraction capability and deep neural architecture for pneumonia classification using imaging data. However, SqueezeViX-Net incorporates a fusion of SqueezeNet, ViT, and Xception modules combined with the StriderOX Adaptive Estimator (SOAE) for dynamic dropout regulation, which is a feature lacking in traditional architecture. The results again are not void of similarities in high accuracy among models; however, SqueezeViX-Net tends to transcend them in precision, recall, and F1-score output due to its adaptive regularization and noise-filtering capabilities. Although all studies point out deep learning as a tool for improved diagnosis of pneumonia, the proposed model draws much stronger conclusions advocating improvement in generalization and robustness across both modalities-X-ray and CT - with statistically significant improvements as demon-strated with ANOVA. Such difference would definitely highlight the strength of SqueezeViX-Net not just in numerical performance but also in terms of architectural flexibility and clinical validity.

Even if the SqueezeViX-Net model on which this paper presents is promising, it has some limitations. First, although the model achieves good accuracy across different imaging modalities, its performance is likely to degrade when variations in imaging protocols and equipment are applied in different medical institutions, potentially affecting its generalizability. Second, although using the dynamic dropout mechanistically through SOAE improves regularization, it causes one additional problem by introducing more complexity in hyperparameter tuning as well as computational overhead during training. The model also relied on a very well-curated and balanced dataset; however, this becomes a limitation to generalization regarding real-world imbalanced or noisy data, which could make the model less effective in uncontrolled clinical settings. Finally, although data were collected using X-rays and CT images, an additional test using other imaging modalities and prospective clinical trials would extend its utility and reliability to standard diagnostic workflows.

The validation and evaluation of the SqueezeViX-Net model in real clinical settings have to consider the complexity and uncertainty of real-world practice settings in medicine. Clinical environments present variations at a much larger and more troubling level, as models can exhibit significantly different performance under these environmental settings. Examples of challenges include varying patient demographics, inconsistent imaging quality, and the presence of comorbidities, all of which affect the model performance. The model demonstrates high accuracy and generalizability in controlled datasets; however, its introduction into the clinical workflow would require extensive prospective validation so as to ascertain its reliability, robustness, and interpretability for the medical personnel given real-time constraints and diagnostic ambiguities.

## 4 DISCUSSIONS

In the present study, the absence of data augmentation should hence be noted as a methodological drawback. Data augmentation has become quite a trend to artificially vary and increase the number of training data through different transformations such as rotation, scaling, translation, flipping, and contrast adjustment, imitating the real-life variations of clinical images. The SqueezeViX-Net with dynamic dropout optimization using SOAE showed promising performance on X-ray and CT datasets. Because of its lesser amount of data augmentation, the model could have missed exposures to wider image variances and real-world perturbations usually encountered within a clinical arena. Therefore, a possible extension in this regard is to test the suggested model's performance against larger datasets following data augmentation procedures. These augmentations must be implemented with extreme care so that the diagnostic feature of the augmented images is preserved while the variability in terms of pixel values, structural appearance, and semantical characteristics is augmented. This could lead, in turn, to sturdy and generalizable model behavior across varying populations and imaging environments. Such a proposal can help increase model performance with unseen data that present vast variability more likely resembling the complexities and uncertainties observed in front-line clinical practice.

## CONCLUSION

This artcile describes SqueezeViX-Net, a pneumonia classifier model that is coherent with a Self-Optimizing Adaptive Estimator (SOAE) dropout for dynamic regulation of its five dropout rates for improved generalization and accuracy. SqueezeViX-Net incorporates a fine-tuned network archi-tecture for medical images, it integrates feature extraction and saliency methods, which localize the disease-related features in X-ray and CT scans. The model is also newly expanded with SOAE, which can explicitly adjust the dropout rates while training and prevent overfitting without compromising performance, a previously unaddressed problem with static dropout. The main novelty of this work is the integration of the proposed SqueezeViX-Net and SOAE models, which achieves the robust image classification with low complexity. This architecture not only improves the extraction of features but also enhances the prominence of these features, thereby improving the resilience of current approaches to pneumonia detection across different image modalities. Furthermore, the proposed scheme of SOAE enables real-time changes and tuning of the model's complexity based on the variability of input data characteristics of CT and X-ray images—a valuable feature when working with CT and X-ray data. Adaptive nature of these assessments promotes enhanced diagnostic concordance and plays to the clinical utility of the model. Regarding the dataset usage and performance, SqueezeViX-Net was based on the pneumonia datasets from Kaggle including the X-ray and CT images that contain various pneumonia type images: bacterial, viral, fungal, and normal kinds of images. All assessed parameters concerning accuracy, precision, recall, and F1 score showed SqueezeViX-Net predominant over existing brands of DenseNet121, ResNet152V2, EfficientNetB7. Such enhancements imply that SqueezeViX-Net has better capability to provide more accurate and reliable classifications from the patterns of medical images. By achieving a significant improvement in training speed and inference time, as well as notable improvements in most evaluation criteria, SqueezeViX-Net demonstrates the potential to be applied in various clinical diagnosis processes, enhancing decision-making and the accuracy of pneumonia differentiation.

With the proposed SqueezeViX-Net model, activities in future work can be extended regarding other crucial medical imaging procedures besides pneumonia classification. Current subjects engaged in this study are X-ray and CT modalities only for identification of pneumonia without full exploration of SqueezeViX-Net potential in other areas. The proposed method has not been applied to different actual modalities in medical imaging, such as ultrasound or MRI. For example, a plethora of existing techniques do classify various conditions such as breast cancer, gliomas, and cervical cancer along with portal and hepatic vessels, but strategically developed derivative forms of SqueezeViX-Net can yield more significant results with much computation efficiency. Its design, coupled with the flexible and much efficient feature extraction mechanism suitable for complex datasets such as mentioned above, under the dynamic dropout adjustment given by SOAE, can be very useful. The next much-enhanced form of SqueezeViX-Net could be adapted towards segmentation tasks, such as the segmentation of liver and kidney structures from abdominal MRI. In such cases, traditional probabilistic and deterministic models may not perform well due to high anatomical variability and noise. Thus, making the data hybrid and dynamic while regularizing techniques would make SqueezeViX-Net suitable to secure the segmentations up on the required performance level. Hence, extending applications of this approach across a wider variety of modalities and diagnoses will be an obviously and prestigious direction for future research.

## Figures and Tables

**Fig. (1):**
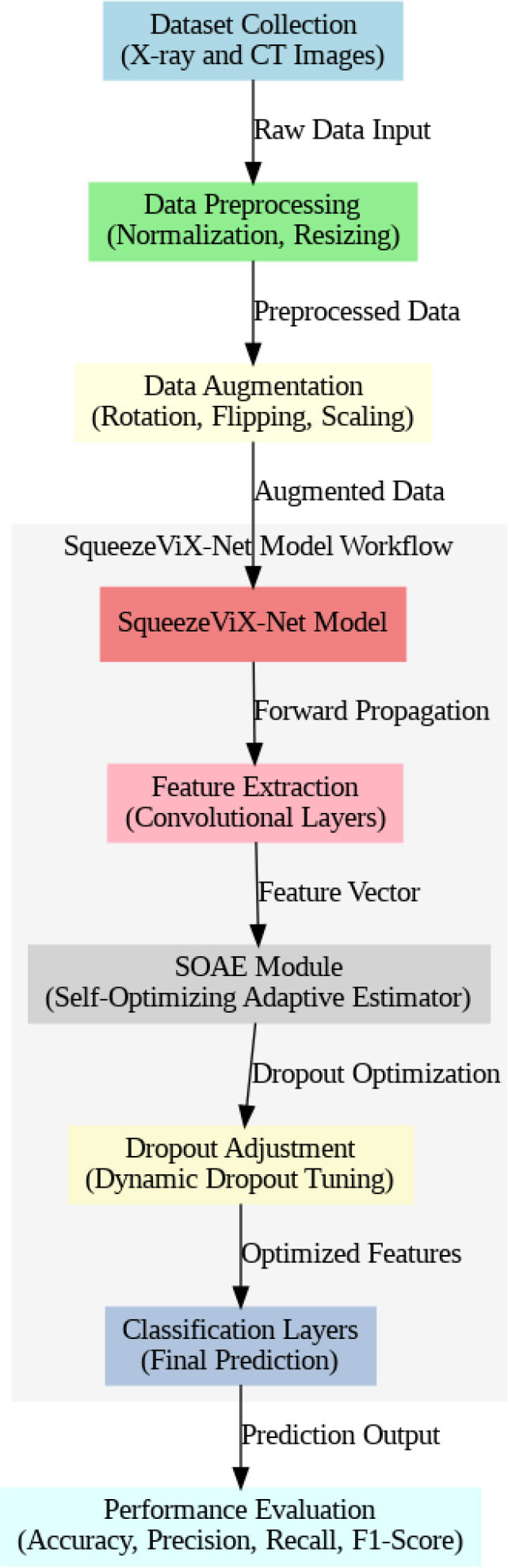
Overall flow of the proposed work.

**Fig. (2):**
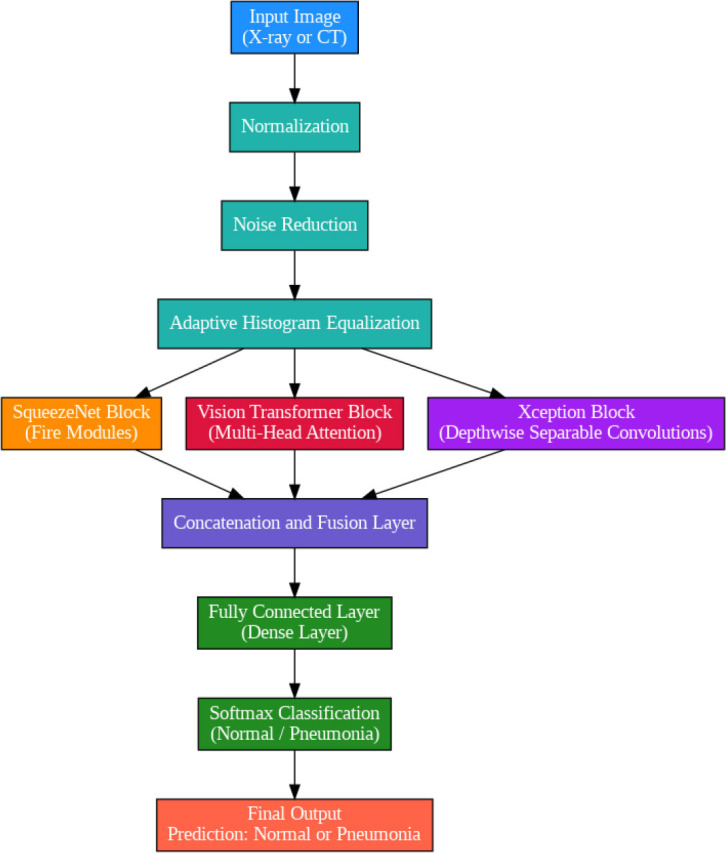
Flow of the proposed squeezeViX-Net model.

**Fig. (3):**
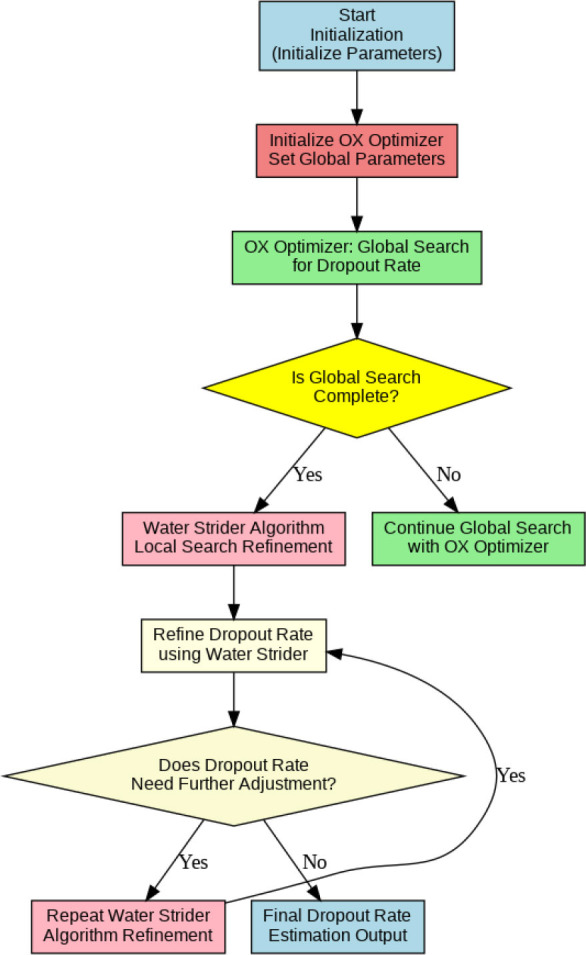
Flow for the proposed striderOX model.

**Fig. (4a, b):**
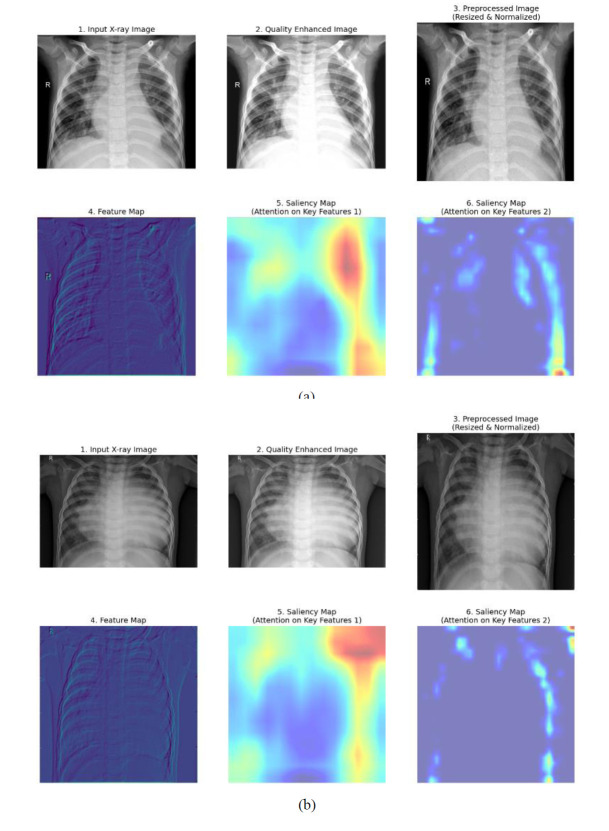
Sample input and output X-Ray images.

**Fig. (5a, b):**
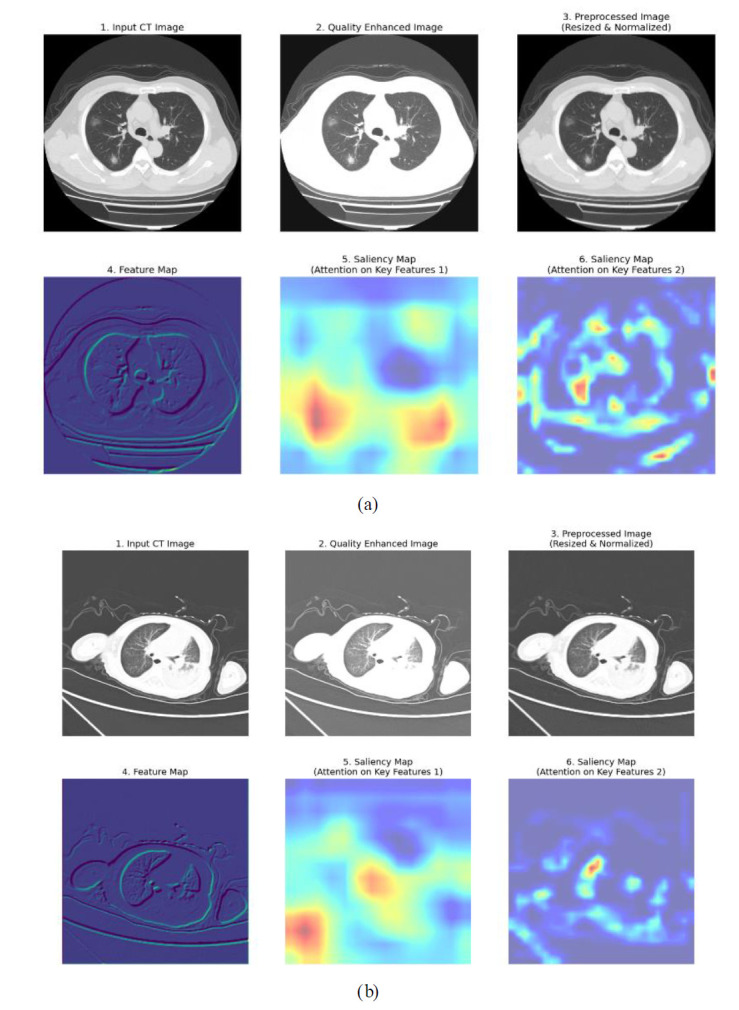
Sample input and output CT images.

**Fig. (6a):**
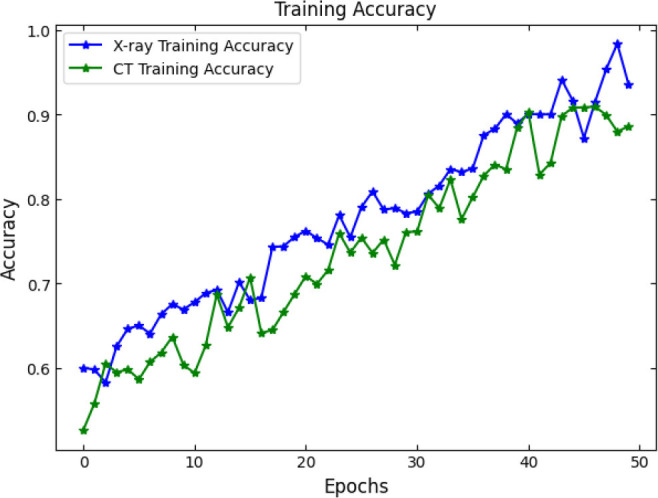
Training accuracy for both X-Ray and CT image dataset.

**Fig. (6b):**
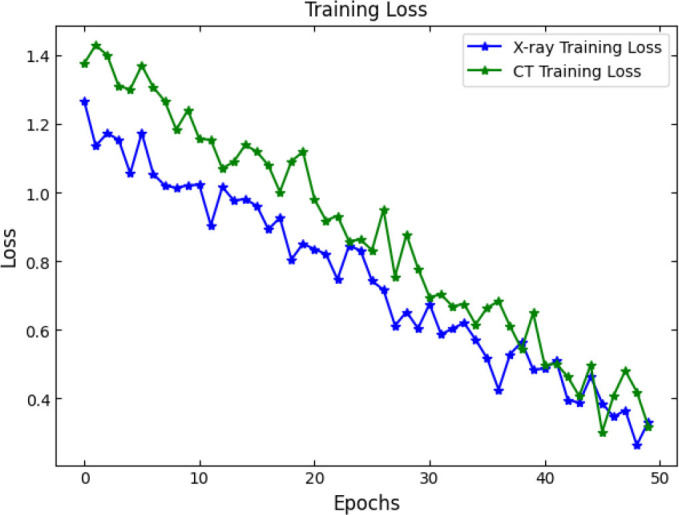
Training loss for both X-Ray and CT image dataset.

**Fig. (7a):**
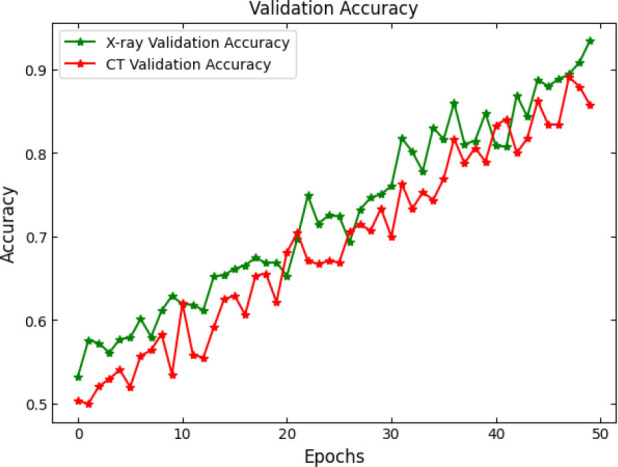
Validation accuracy for both X-Ray and CT image datasets.

**Fig. (7b):**
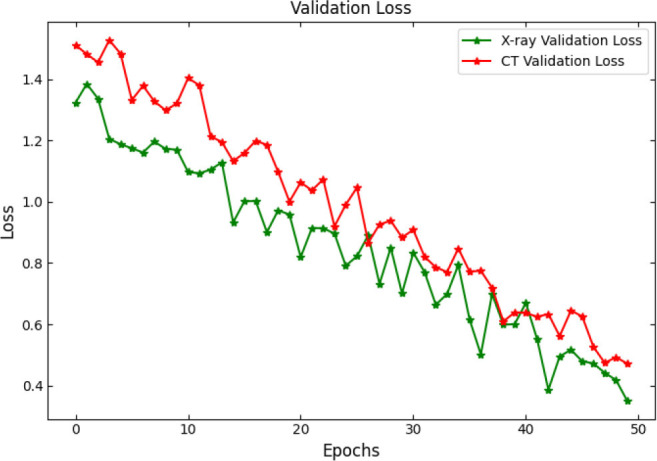
Validation loss for both X-Ray and CT image datasets.

**Fig. (8):**
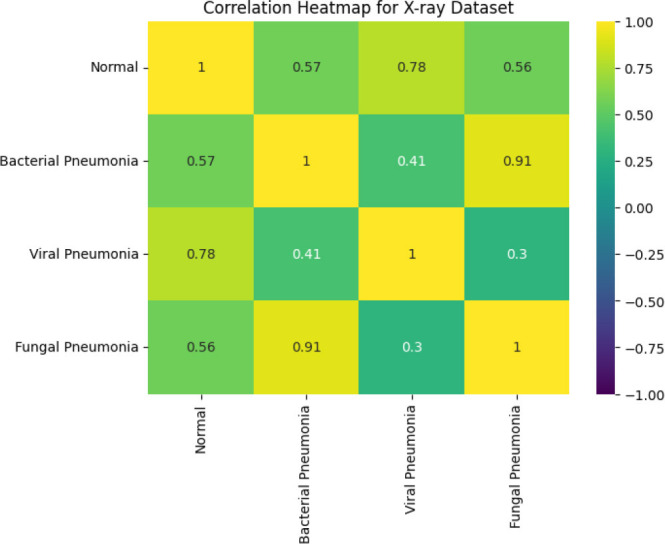
Correlation heatmap for X-Ray dataset.

**Fig. (9):**
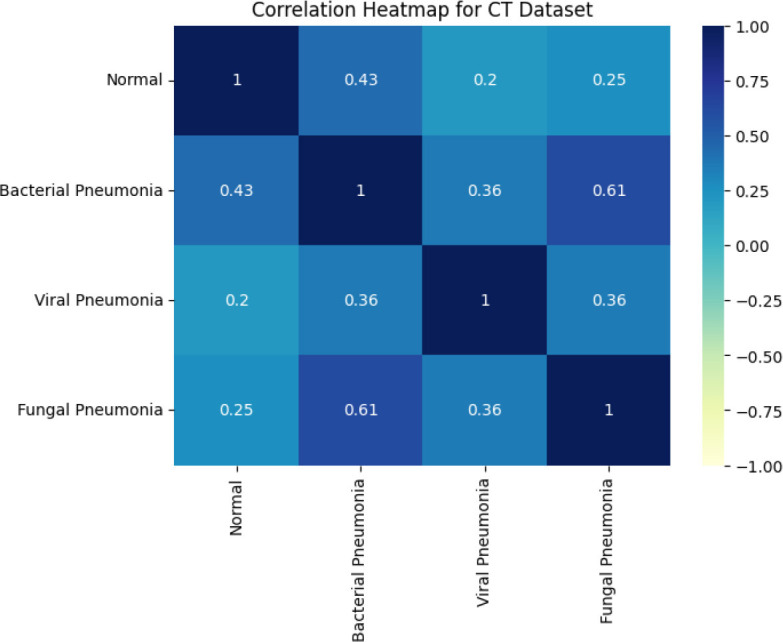
Correlation heatmap for CT dataset.

**Fig. (10):**
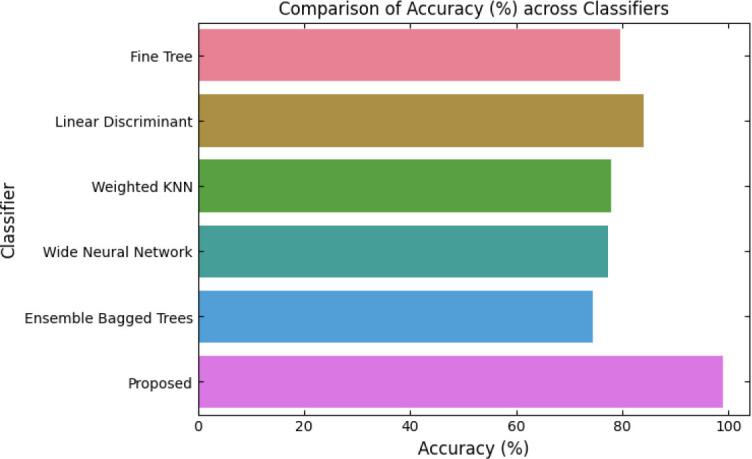
Comparison of accuracy among different classifiers using X-Ray image dataset.

**Fig. (11):**
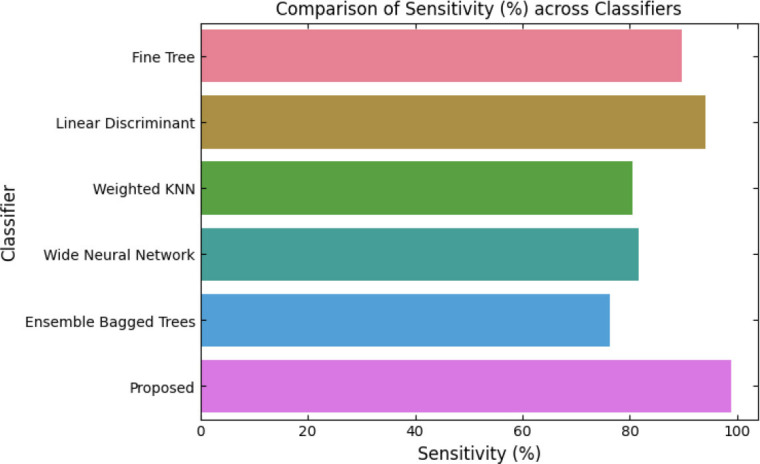
Comparison of sensitivity among different classifiers using the X-Ray image dataset.

**Fig. (12):**
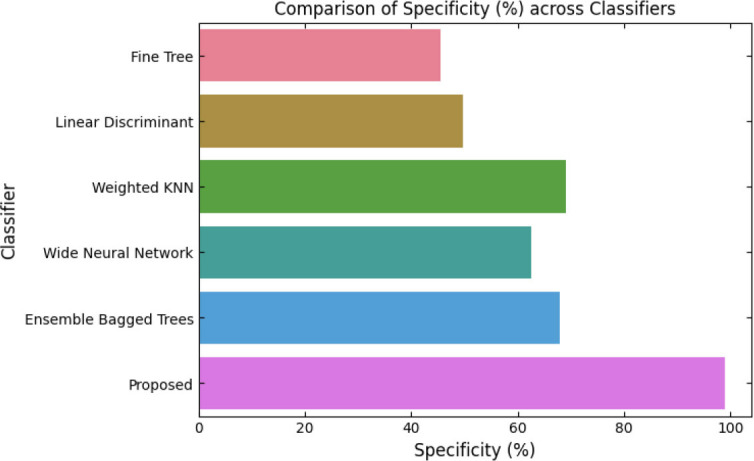
Comparison of specificity among different classifiers using X-Ray image dataset.

**Fig. (13):**
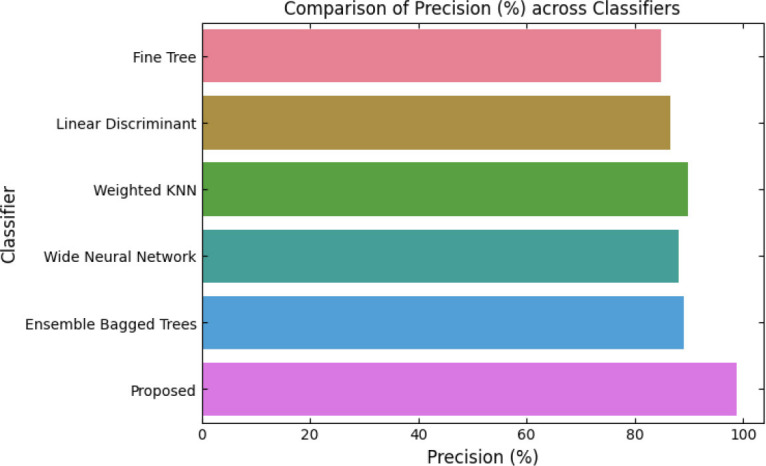
Comparison of precision among different classifiers using X-Ray image dataset.

**Fig. (14):**
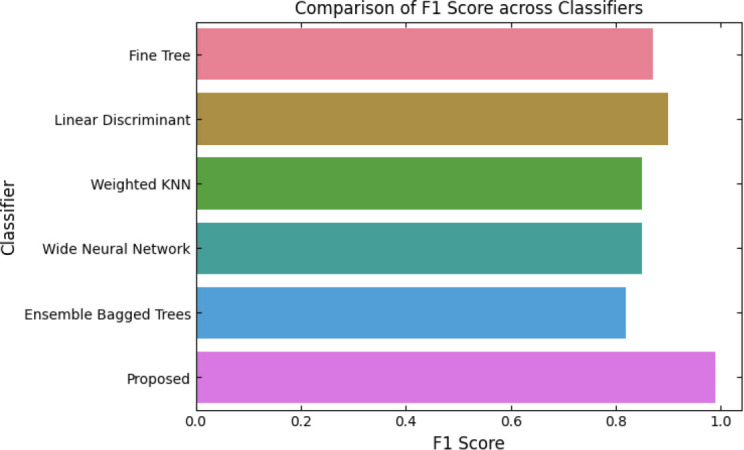
Comparison of the f1-score among different classifiers using the X-Ray image dataset.

**Fig. (15):**
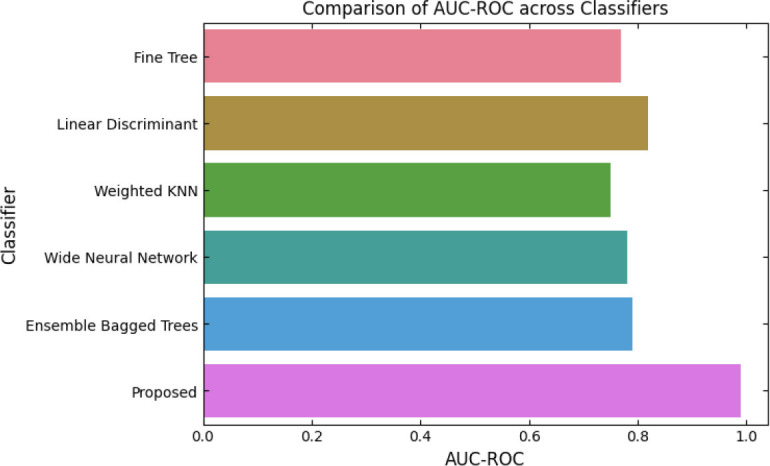
Comparison of AUC-ROC among different classifiers using X-Ray image dataset.

**Fig. (16):**
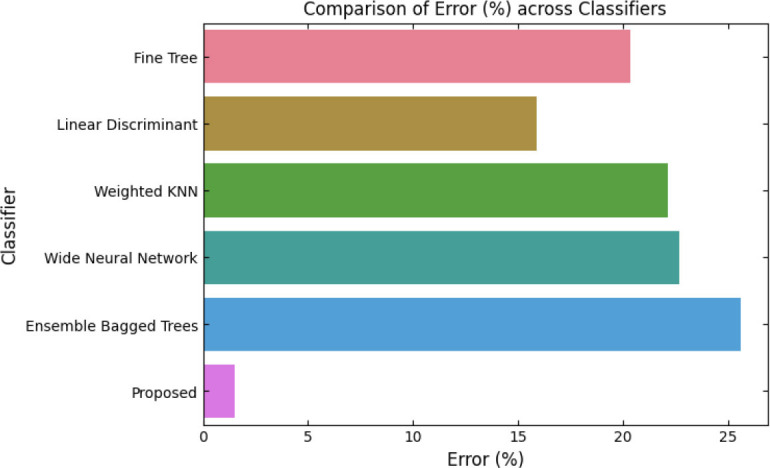
Comparison of error rate among different classifiers using X-Ray image dataset.

**Fig. (17):**
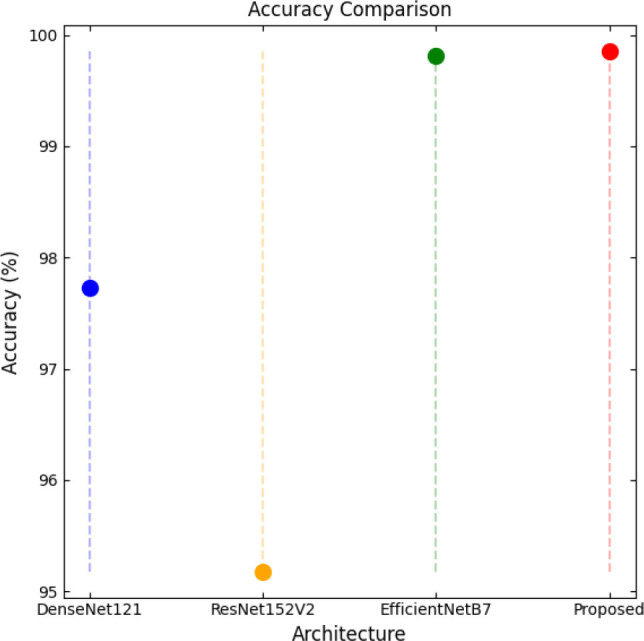
Comparison of accuracy with recent architecture models.

**Fig. (18):**
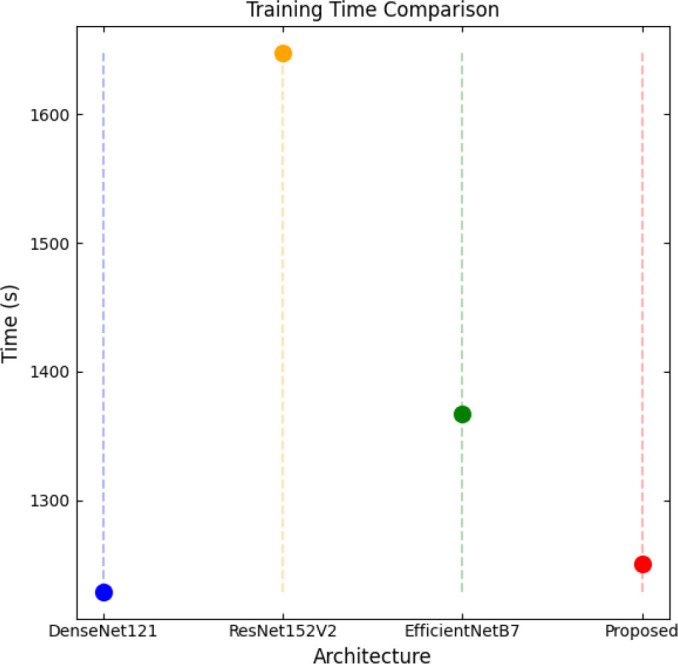
Comparison of training time with recent architecture models.

**Fig. (19):**
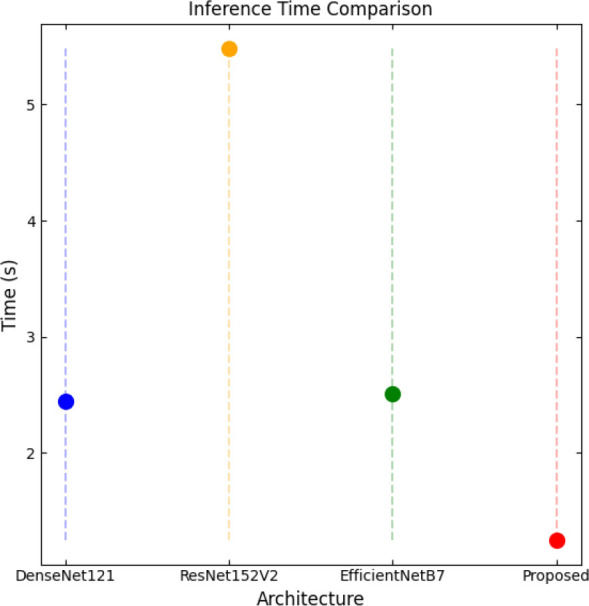
Comparison of inference time with recent architecture models.

**Fig. (20):**
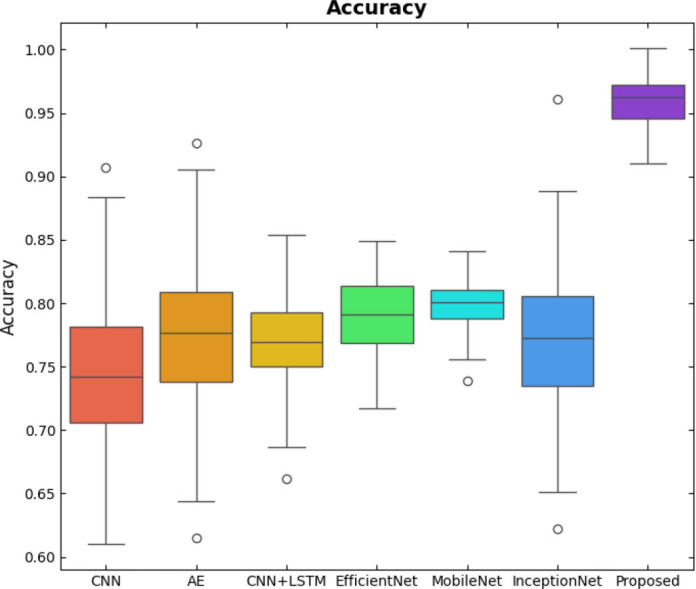
Comparison of accuracy with other deep learning classifiers using the X-Ray dataset.

**Fig. (21):**
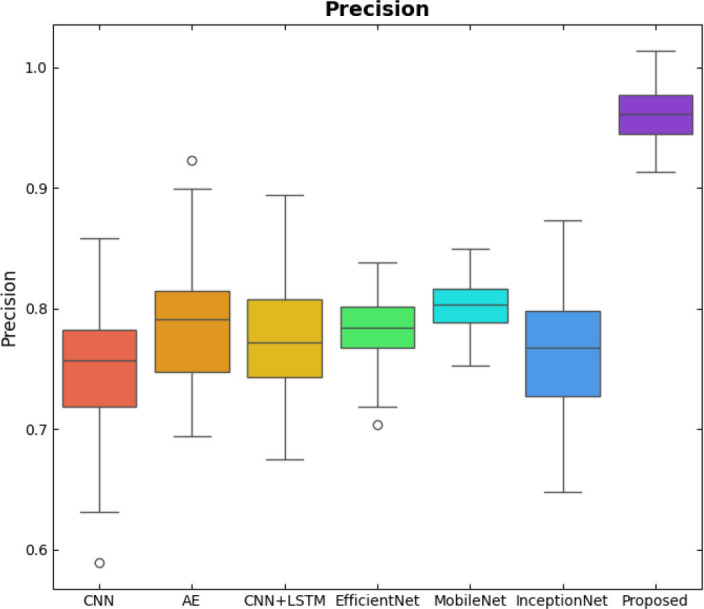
Comparison of precision with other deep learning classifiers using the X-Ray dataset.

**Fig. (22):**
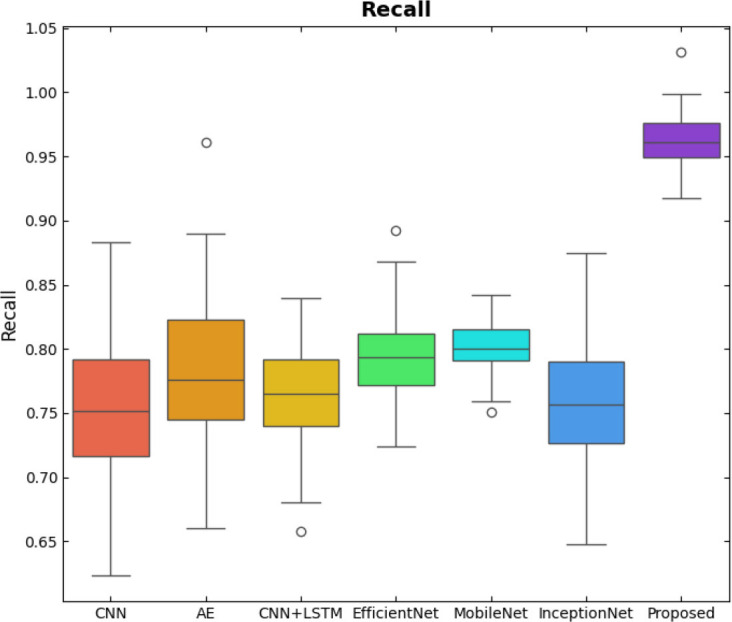
Comparison of recall with other deep learning classifiers using X-Ray dataset.

**Fig. (23):**
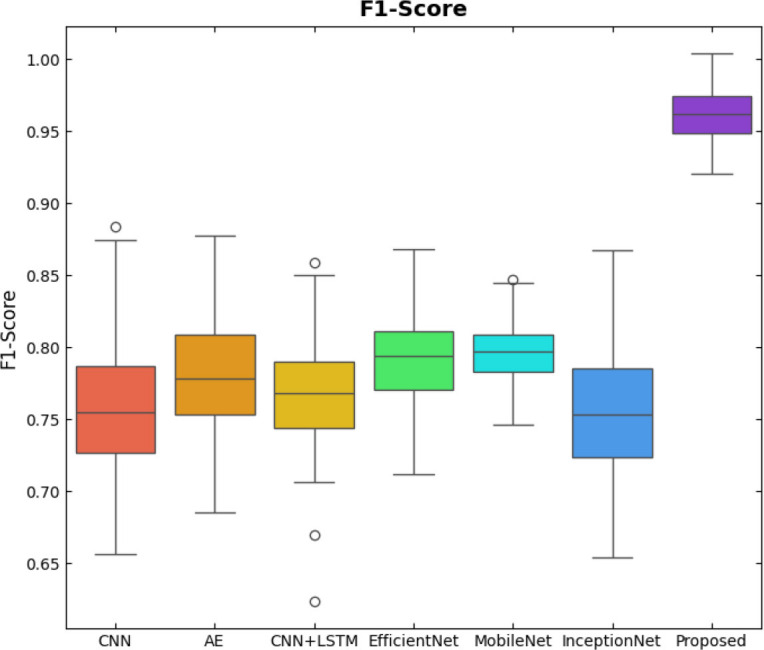
Comparison of f1-score with other deep learning classifiers using X-Ray dataset.

**Fig. (24):**
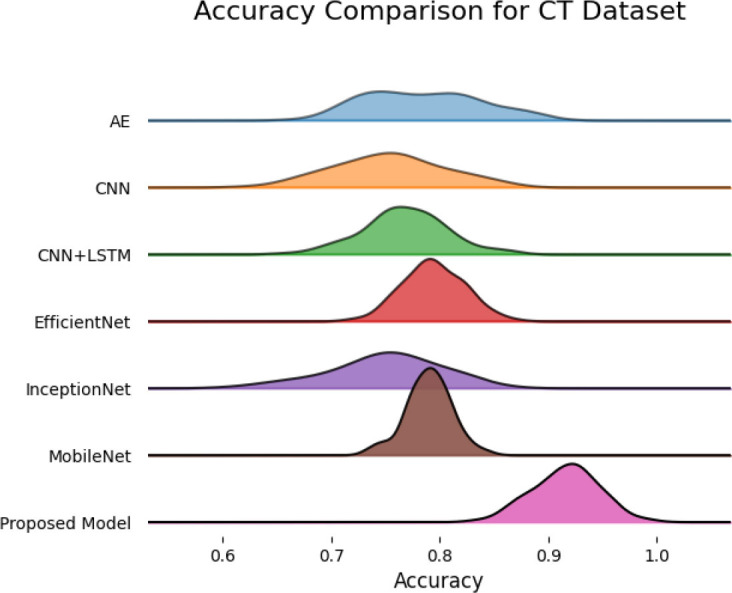
Comparison of accuracy with other deep learning classifiers using the CT dataset.

**Fig. (25):**
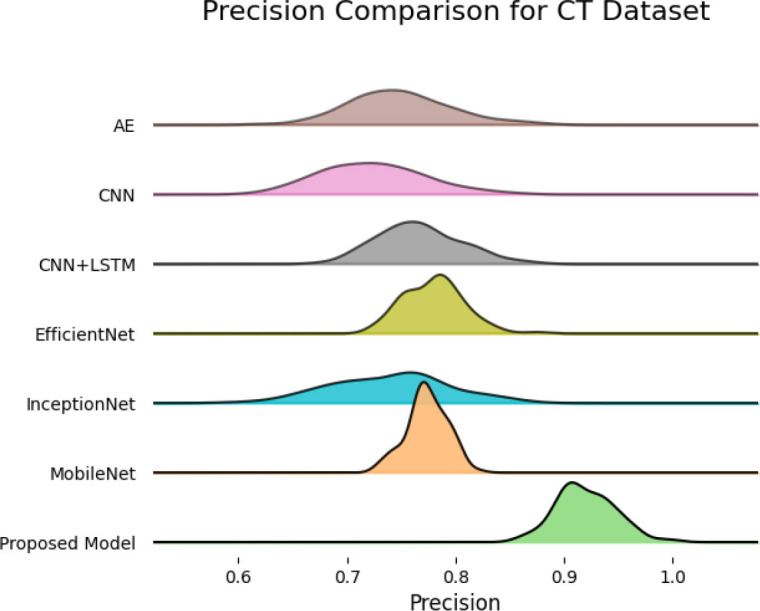
Comparison of precision with other deep learning classifiers using the CT dataset.

**Fig. (26):**
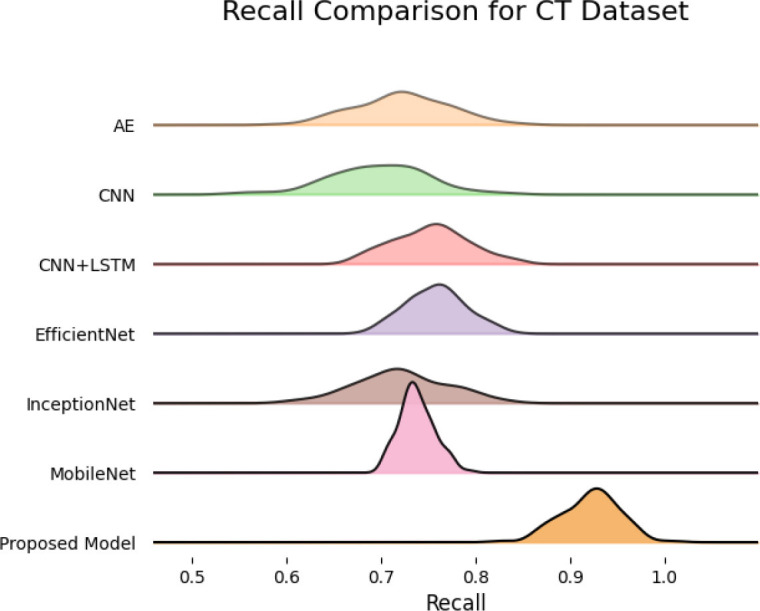
Comparison of recall with other deep learning classifiers using the CT dataset.

**Fig. (27):**
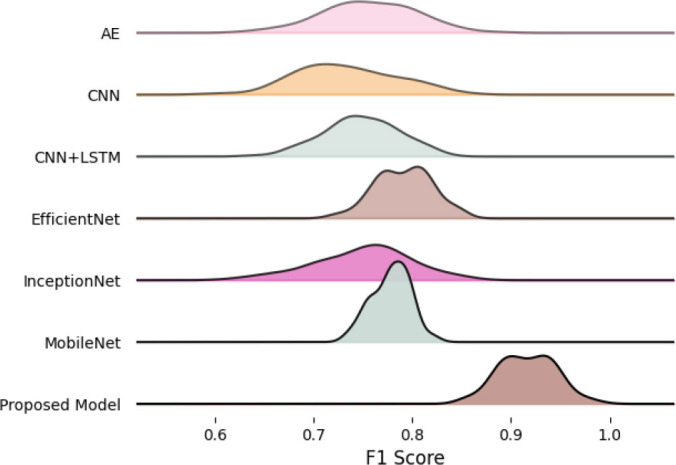
Comparison of the f1-score with other deep learning classifiers using the CT dataset.

**Fig. (28):**
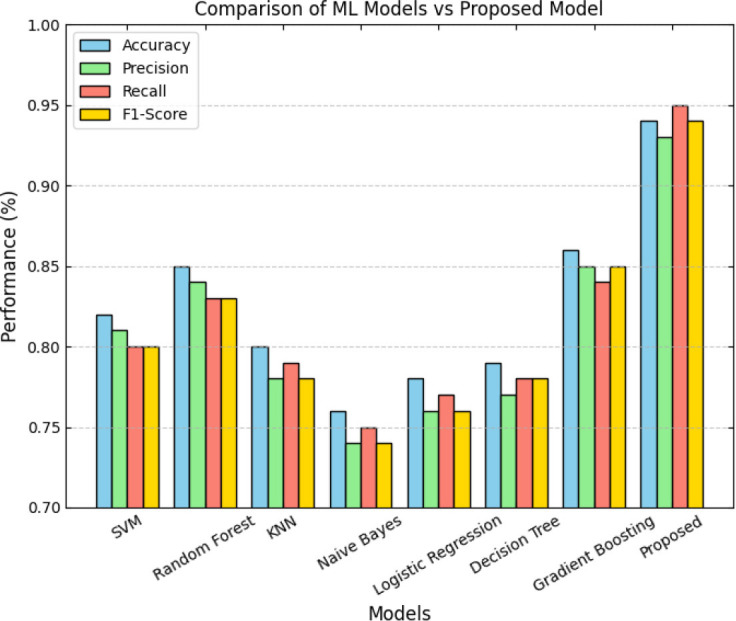
Overall comparative study with traditional deep learning models.

**Table 1:** Statistical test results.

** Component **	** Sum of Squares (SS) **	** Degrees of Freedom (df) **	** Mean Square (MS) **	** F-Value **	***p*-value **
Between Groups	0.143	7	0.02043	52.07	< 0.0001
Within Groups	0.0187	16	0.00117	-	-
** Total **	** 0.1617 **	** 23 **	-	-	-

## Data Availability

All data generated or analyzed during this study are included in this published article.

## LIST OF ABBREVIATIONS

SOAE: Self-Optimized Adaptive Enhancement
CT: Computed Tomography
CAD: Computer-Aided Diagnosis
AMR: Antimicrobial Resistance
KNN: K-Nearest Neighbor
ViT: Vision Transformers
DL: Deep Learning
ML: Machine Learning
CNN: Convolutional Neural Network
LSTM: Long Short-Term Memory
AE: Auto Encoder
NB: Naïve Bayes
LR: Logistic Regression
MS: Mean Square
RF: Random Forest
DT: Decision Tree
GB: Gradient Boost
ResNet: Residual Network

## References

[1] Ahmed M.S., Rahman A., AlGhamdi F., AlDakheel S., Hakami H., AlJumah A., AlIbrahim Z., Youldash M., Alam Khan M.A., Basheer Ahmed M.I. (2023). Joint diagnosis of pneumonia, COVID-19, and tuberculosis from chest X-ray images: A deep learning approach.. Diagnostics.

[2] Akbulut Y. (2023). Automated pneumonia based lung diseases classification with robust technique based on a customized deep learning approach.. Diagnostics.

[3] Humphries S.M., Thieke D., Baraghoshi D., Strand M.J., Swigris J.J., Chae K.J., Hwang H.J., Oh A.S., Flaherty K.R., Adegunsoye A., Jablonski R., Lee C.T., Husain A.N., Chung J.H., Strek M.E., Lynch D.A. (2024). Deep learning classification of usual interstitial pneumonia predicts outcomes.. Am. J. Respir. Crit. Care Med..

[4] Rundo L., Militello C. (2024). Image biomarkers and explainable AI: handcrafted features *versus* deep learned features.. Eur Radiol Exp.

[5] Arulananth T.S., Prakash S.W., Ayyasamy R.K., Kavitha V.P., Kuppusamy P.G., Chinnasamy P. (2024). Classification of paediatric pneumonia using modified DenseNet-121 deep-learning model.. IEEE Access.

[6] Asnake N.W., Salau A.O., Ayalew A.M. (2024). X-ray image-based pneumonia detection and classification using deep learning.. Multimedia Tools Appl..

[7] Ali Z., Khan M.A., Hamza A., Alzahrani A.I., Alalwan N., Shabaz M., Khan F. (2024). A deep learning-based x-ray imaging diagnosis system for classification of tuberculosis, COVID-19, and pneumonia traits using evolutionary algorithm.. Int. J. Imaging Syst. Technol..

[8] Parthasarathy V., Saravanan S. (2024). Computer aided diagnosis using Harris Hawks optimizer with deep learning for pneumonia detection on chest X-ray images.. Int. J. Inf. Technol..

[9] Rodríguez A., Tabassum A., Cui J., Xie J., Ho J., Agarwal P. (2021). Deepcovid: An operational deep learning-driven framework for explainable real-time covid-19 forecasting.. Proc. AAAI Conf. Artif. Intell..

[10] Lasker A., Ghosh M., Obaidullah S.M., Chakraborty C., Roy K. (2023). LWSNet: A novel deep-learning architecture to segregate Covid-19 and pneumonia from x-ray imagery.. Multimedia Tools Appl..

[11] Wu L., Zhang J., Wang Y., Ding R., Cao Y., Liu G., Liufu C., Xie B., Kang S., Liu R., Li W., Guan F. (2024). Pneumonia detection based on RSNA dataset and anchor-free deep learning detector.. Sci. Rep..

[12] Wang T., Nie Z., Wang R., Xu Q., Huang H., Xu H., Xie F., Liu X.J. (2023). PneuNet: deep learning for COVID-19 pneumonia diagnosis on chest X-ray image analysis using Vision Transformer.. Med. Biol. Eng. Comput..

[13] Wen R., Xu P., Cai Y., Wang F., Li M., Zeng X., Liu C. (2023). A deep learning model for the diagnosis and discrimination of gram-positive and gram-negative bacterial pneumonia for children using chest radiography images and clinical information.. Infect. Drug Resist..

[14] Chung J.H., Chelala L., Pugashetti J.V., Wang J.M., Adegunsoye A., Matyga A.W., Keith L., Ludwig K., Zafari S., Ghodrati S., Ghasemiesfe A., Guo H., Soo E., Lyen S., Sayer C., Hatt C., Oldham J.M. (2024). A deep learning-based radiomic classifier for usual interstitial pneumonia.. Chest.

[15] Ganeshkumar M., Ravi V., Sowmya V., Gopalakrishnan E.A., Soman K.P., Rupeshkumar M. (2023). Two-stage deep learning model for automate detection and classification of lung diseases.. Soft Comput..

[16] Bal U., Bal A., Moral Ö.T., Düzgün F., Gürbüz N. (2024). A deep learning feature extraction-based hybrid approach for detecting pediatric pneumonia in chest X-ray images.. Phys. Eng. Sci. Med..

[17] Celik G. (2023). Detection of Covid-19 and other pneumonia cases from CT and X-ray chest images using deep learning based on feature reuse residual block and depthwise dilated convolutions neural network.. Appl. Soft Comput..

[18] Gill K.S., Anand V., Chauhan R., Rawat D., Gupta R. Using deep learning and mobilenet50v2 cnn model to classify chest x-ray images for pneumonia disease detection.. 2023 2nd International Conference on Futuristic Technologies (INCOFT).

[19] Ali M., Shahroz M., Akram U., Mushtaq M.F., Altamiranda S.C., Obregon S.A., Díez I.D.L.T., Ashraf I. (2024). Pneumonia detection using chest radiographs with novel efficientNetV2L model.. IEEE Access.

[20] Mohammadian Takaloo V., Hashemzadeh M., Ghavidel Neycharan J. (2024). DiagCovidPNA: diagnosing and differentiating COVID-19, viral and bacterial pneumonia from chest X-ray images using a hybrid specialized deep learning approach.. Soft Comput..

[21] Quispe S., Arellano I., Shiguihara P. (2023). A survey of deep learning techniques based on computed tomography images for detection of pneumonia.. Eng. Proc..

[22] Hassan F., Mehmood M.H., Rahman A.U., Khan W., Khalid S., Ali M. ViBaNet: A novel deep learning approach to detect bacterial and viral pneumonia.. 2023 International Conference on Frontiers of Information Technology (FIT).

[23] Ghnemat R., Alodibat S., Abu Al-Haija Q. (2023). Explainable Artificial Intelligence (XAI) for deep learning based medical imaging classification.. J. Imaging.

[24] Ali M.U., Zafar A., Tanveer J., Khan M.A., Kim S.H., Alsulami M.M., Lee S.W. (2024). Deep learning network selection and optimized information fusion for enhanced COVID-19 detection.. Int. J. Imaging Syst. Technol..

[25] Riedel P., von Schwerin R., Schaudt D., Hafner A., Späte C. (2023). ResNetFed: federated deep learning architecture for privacy-preserving pneumonia detection from COVID-19 chest radiographs.. J. Healthc. Inform. Res..

[26] Vetrithangam D. (2023). Prediction of pneumonia disease from x-ray images using a modified ResNet152V2 deep learning model.. J Theor Appl Inf Technol.

[27] Reshan M.S.A., Gill K.S., Anand V., Gupta S., Alshahrani H., Sulaiman A. (2023). Detection of pneumonia from chest X-ray images utilizing MobileNet model.. Healthcare.

[28] Udbhav M., Attri R.K., Vijarania M., Gupta S., Tripathi K. (2023). Pneumonia detection using chest X-ray with the help of deep learning.. Concepts of Artificial Intelligence and its Application in Modern Healthcare Systems.

[29] Arora N., Kakde A., Sharma S.C. (2023). An optimal approach for content-based image retrieval using deep learning on COVID-19 and pneumonia X-ray Images.. Int. J. System. Assurance. Eng. Manag..

[30] Ibrahim A.U., Ozsoz M., Serte S., Al-Turjman F., Yakoi P.S. (2021). Pneumonia classification using deep learning from chest X-ray images during COVID-19.. Cognit. Comput..

[31] Wang F., Li X., Wen R., Luo H., Liu D., Qi S., Jing Y., Wang P., Deng G., Huang C., Du T., Wang L., Liang H., Wang J., Liu C. (2023). Pneumonia-Plus: A deep learning model for the classification of bacterial, fungal, and viral pneumonia based on CT tomography.. Eur. Radiol..

[32] Sharma S., Guleria K. (2023). A systematic literature review on deep learning approaches for pneumonia detection using chest X-ray images.. Multimedia Tools Appl..

[33] Goyal S., Singh R. (2023). Detection and classification of lung diseases for pneumonia and Covid-19 using machine and deep learning techniques.. J. Ambient Intell. Humaniz. Comput..

[34] Sharma S., Guleria K. (2023). A deep learning based model for the detection of pneumonia from chest X-ray images using VGG-16 and neural networks.. Procedia Comput. Sci..

[35] Sharma S., Guleria K. (2023). A deep learning model for early prediction of pneumonia using VGG19 and neural networks.. Mobile Radio Communications and 5G Networks.

[36] Yi R., Tang L., Tian Y., Liu J., Wu Z. (2023). Identification and classification of pneumonia disease using a deep learning-based intelligent computational framework.. Neural Comput. Appl..

[37] Sanghvi H.A., Patel R.H., Agarwal A., Gupta S., Sawhney V., Pandya A.S. (2023). A deep learning approach for classification of COVID and pneumonia using DenseNet -201.. Int. J. Imaging Syst. Technol..

[38] Siddiqi R., Javaid S. (2024). Deep learning for pneumonia detection in chest x-ray images: A comprehensive survey.. J. Imaging.

[39] Ibrahim D. M., Elshennawy N. M., Sarhan A. M. (2021). Deep-chest: Multi-classification deep learning model for diagnosing COVID-19, pneumonia, and lung cancer chest diseases.. J. Theor. Appl. Inf Technol.

[40] Moussaid A., Zrira N., Benmiloud I., Farahat Z., Karmoun Y., Benzidia Y. (2023). On the implementation of a post-pandemic deep learning algorithm based on a hybrid ct-scan/x-ray images classification applied to pneumonia categories.. Healthcare.

